# Supplementary material for: A single atom change turns insulating saturated wires into molecular conductors
Source: Nat Commun. 2021 Jun 8;12:3432. doi: 10.1038/s41467-021-23528-8 (PMC8187423; doi:10.1038/s41467-021-23528-8)
Supplement: Supplementary file 1 — Supplementary Information [file 41467_2021_23528_MOESM1_ESM.docx]

Supplementary Information for

**A Single Atom Change Turns Insulating Saturated Wires into Molecular Conductors**

*Xiaoping Chen^1,2^, Bernhard Kretz^3^, Francis Adoah^4^, Cameron Nickle^4^, Xiao Chi^5^, Xiaojiang Yu^5^, Enrique del Barco^4^, Damien Thompson^6^, David A. Egger^3^*,* and *Christian A. Nijhuis^1,2^**

^1^Department of Chemistry, National University of Singapore, 3 Science Drive 3, Singapore 117543, Singapore.

^2^Centre for Advanced 2D Materials and Graphene Research Centre, National University of Singapore, 6 Science Drive 2, Singapore 117546, Singapore.

^3^Department of Physics, Technical University of Munich, 85748 Garching, Germany

^4^Department of Physics, University of Central Florida, Orlando, Florida 32816 - USA

^5^Singapore Synchrotron Light Source, National University of Singapore, 5 Research Link, Singapore 117603, Singapore.

^6^Department of Physics, Bernal Institute, University of Limerick, V94 T9PX, Ireland

Correspondence to: chmnca@nus.edu.sg; david.egger@tum.de

**Supplementary Section 1: Materials and Methods.**

**Materials**

All the regents and chemicals were purchased from Sigma-Aldrich or Alfa Aesar unless states otherwise. Chemicals and solvents were directly used without further purification or treatment. 1, 14-Dibromotetradecane was purchased from Apollo Scientific Ltd.  14-Bromo-1-Tetradecanol was purchased from Fluorochem Ltd. 16-Bromohexadecanol was purchased from ASTATECH, INC. 18-Bromo-1-Octadecene was purchased from Tokyo Chemical Industry CO., Ltd. Deionized water was prepared with an Elga Purelab option-Q system. Silica gel (60Å/40-63 µm) was obtained from Sigma-Aldrich. ^1^H and ^13^C NMR spectra were recorded from a Bruker Avance 300 MHz spectrometer or a Bruker Avance 400 MHz spectrometer with CDCl_3_ as solvent. Electron ionization (EI) mass spectra were collected with a Finnigan LCQ mass spectrometer. High resolution electron ionization mass spectra (EI-HR-MS) were recorded from a Bruker microTOF-QII mass spectrometer. The Ag (purity: 99.99 %) was purchased from MOS Group Pte Ltd (Singapore). The 6 inch Prime Si wafers (orientation 100) were purchased from SYST Integration Pte Ltd (Singapore). The thermal adhesive was purchased from Pacific Adhesive System (M) Sdn, Bhd. (Malaysia). The ethanol (assay: 99.94 % in V/V) was purchased from VWR Chemicals (France) and distilled for SAM incubation.

**Supplementary Section 2: Synthetic Procedures.**

**Synthesis Scheme**

Supplementary Fig.1 shows the synthetic routes of the HS(CH_2_)*_n_*X SAM precursors. The synthetic methods for *n* = 11 have been reported before.^1^ Therefore, we generally followed this procedure (Supplementary Fig. 1a-d) for our molecules except for *n* = 16 and 18 as indicated in Supplementary Fig. 1.

**Supplementary Fig. 1.** The synthetic scheme of the HS(CH_2_)*_n_*X compounds.

**10-fluorodecane-1-thiol.** We synthesized 1-bromo-10-fluorodecane according to a procedure reported in the literature (Supplementary Fig. 1a).^2^ 10-bromo-1-decanol (8.00 g, 33.7 mmol), 100 mL distilled DCM, and pyridine (3.12 mL, 38.7 mmol) were added into a two-neck flask under N_2_ and cooled with salty ice water. The trifluoromethanesulfonic anhydride, Tf_2_O (10.00 g, 35.4 mmol) was added dropwise. The mixture was stirred for another 30 min, then washed with 1N HCl. The organic layer was separated and dried over anhydrous Na_2_SO_4_. Tetrabutylammonium hydrogen difluoride, TBAHF (15 mL, 36 mmol) was added to the filtrate and the mixture was further stirred for 2 h. Then, the solvent was removed under reduced pressure and the residue was extracted with hexane three times. The extracts were combined and concentrated. The product 1-bromo-10-fluorodecane (3.20 g, yield 40.2 %) was collected by column chromatography (second fraction) using hexane as eluent. ^1^H-NMR (400 MHz, CDCl_3_): δ 1.29-1.40 (m, 12H, -*(CH_2_)_6_*-CH_2_CH_2_F), 1.64-1.71(m, 2H, -*CH_2_*-CH_2_F), 1.84 (p, *J* =6.8 Hz, 2H, -*CH_2_*-CH_2_Br), 3.39 (t, *J* =6.8 Hz, 2H, -*CH_2_*-Br), 4.42 (dt, *J*_HF_ =47.6 Hz, *J*_HH_ =6.4 Hz, 2H, -*CH_2_*-F) ppm. ^13^C-NMR (100 MHz, CDCl_3_): δ = 25.25 (d, ^3^*J*_CF_ = 5.7 Hz, -*C*-C-C-F), 28.25, 28.83, 29.29, 29.42, 29.48, 30.51 (d, ^2^*J*_CF_ = 19.3 Hz, -*C*-C-F), 32.93, 34.00, 84.22 (d, ^1^*J*_CF_ = 163.3 Hz, -*C*-F) ppm. EI-MS: 240 (M^+^).

The 1-fluoro-10-thiocyanatodecane was synthesized following a reported method.^3^ Potassium thiocyanate (0.48 g, 5.0 mmol) was dissolved in 20 mL ethanol, the mixture was refluxed, to which 1-bromo-10-fluorodecane (0.80 g, 3.3 mmol) in 10 mL ethanol was added slowly in 20 min. The mixture was allowed to reflux for another 3 h before cooling down to room temperature. The white precipitate was filtered and the solvent was removed. We dissolved the residue in DCM followed by washing with DI water, drying over anhydrous Na_2_SO_4_ and then concentrated. The raw product was purified by column chromatography with ethyl acetate : hexane = 1 : 20. The yield of this step was 70 %. ^1^H-NMR (400 MHz, CDCl_3_): δ 1.30-1.41 (m, 12H, -*(CH_2_)_6_*-CH_2_CH_2_F), 1.64-1.71 (m, 2H, -*CH_2_*-CH_2_F), 1.81 (p, *J* =7.2 Hz, 2H, -*CH_2_*-CH_2_SCN), 2.93 (t, *J* =7.2 Hz, 2H, -*CH_2_*-SCN), 4.42 (dt, *J*_HF_ =47.6 Hz, *J*_HH_ =6.4 Hz, 2H, -*CH_2_*-F) ppm. ^13^C-NMR (100 MHz, CDCl_3_): δ = 25.21 (d, ^3^*J*_CF_ = 5.6 Hz, -*C*-C-C-F), 27.99, 28.90, 29.22, 29.30, 29.41, 29.95, 30.46 (d, ^2^*J*_CF_ = 19.3 Hz, -*C*-C-F), 34.14, 84.17 (d, ^1^*J*_CF_ = 163.2 Hz, -*C*-F), 112.43 (-S-*C*≡N) ppm. EI-MS: 216 (M^+^).

The 10-fluorodecane-1-thiol was prepared according to a literature method with minor modifications.^3^ Lithium ammonium anhydride (0.23 g, 6.8 mmol) was added into a two-neck flask with 20 mL distilled diethyl ether under N_2_ at 0 °C, to which 1-fluoro-10-thiocyanatodecane (0.74 g, 3.4 mmol) in 5 mL distilled diethyl ether was added dropwise. The mixture was allowed to react for 10 min. After that, degassed water was added to quench the reaction. Then, 3 N HCl was added until the suspension became clear, after which the aqueous solution was extracted with diethyl ether 3 times. The organic layers were combined and washed with DI water for 3 times, dried over anhydrous Na_2_SO_4_ and then concentrated. The product was found in the first band by column chromatography with hexane as the eluent, yield 88 % (0.58 g, 3.0 mmol). ^1^H-NMR (400 MHz, CDCl_3_): δ 1.29-1.39 (m, 12H, -*(CH_2_)_6_*-CH_2_CH_2_F), 1.61-1.72 (m, 4H, -*CH_2_*-CH_2_F and -*CH_2_*-CH_2_SH), 2.52 (q, *J* =7.2 Hz, 2H, -*CH_2_*-SH), 4.43 (dt, *J*_HF_ =47.6 Hz, *J*_HH_ =6.4 Hz, 2H, -*CH_2_*-F) ppm. ^13^C-NMR (75 MHz, CDCl_3_): δ = 24.75, 25.28 (d, ^3^*J*_CF_ = 5.5 Hz, -*C*-C-C-F), 28.48, 29.16, 29.33, 29.52, 29.55, 30.55 (d, ^2^*J*_CF_ = 19.3 Hz, -*C*-C-F), 34.16, 84.30 (d, ^1^*J*_CF_ = 163.2 Hz, -*C*-F) ppm. EI-MS: 192 (M^+^). Melting point: -21 ℃.

**10-chlorodecane-1-thiol**. 1,10-diiododecane was ordered from Sigma-Aldrich directly. Here we give the synthesis of 1,12-diiodododecane as an example. 6.20 g (18.9 mmol) 1,12-dibromododecane and 9.60 g (64.1 mmol) sodium iodine were dissolved in 150 mL acetone and refluxed for 5 h. After cooling down to room temperature, the solvent was removed by rotary evaporator. Diethylether was used to dissolve the residue and the undissolved precipitate was filtered, the organic mixture was washed 3 times with water, dried over anhydrous Na_2_SO_4_ and then concentrated. The concentrated solid was as such for the next step, yield 95.2 % (7.60 g, 18.0 mmol). ^1^H-NMR (300 MHz, CDCl_3_): δ 1.27-1.39 (m, 16H, -(*CH_2_)_8_*-CH_2_CH_2_I), 1.81 (p, *J* =7.2 Hz, 4H, -*CH_2_*-CH_2_I), 3.18 (t, *J* =7.2 Hz, 4H, -*CH_2_*-I) ppm. ^13^C-NMR (75 MHz, CDCl_3_): δ =7.44 (-*C*-I), 28.69, 29.55, 29.67, 29.72, 30.66, 33.73 ppm. EI-MS: 422 (M^+^).

10-iodo-1-decanethioacetate was synthesized using a similar method to that reported by Alvarado et al.^4^ We dissolved 1,10-diiododecane (6.00 g, 15.2 mmol) in 200 mL acetonitrile and refluxed the mixture. 1.73 g, 15.1 mmol potassium thioacetate was added to the mixture in three batches at intervals of 1.5 hours. After the reaction was finished, the mixture was allowed to cool down to room temperature. The solvent was removed and the residue was picked up by DCM and washed three times with DI water. The organic layer was dried over anhydrous Na_2_SO_4_ and then concentrated. The obtained solid was then purified by column chromatography with hexane and DCM at a volume ratio of 10:1 with a yield of 33.2 % (1.73 g, 5.05 mmol). ^1^H-NMR (300 MHz, CDCl_3_): δ 1.27-1.36 (m, 12H, -(*CH_2_)_6_*-CH_2_CH_2_I), 1.55 (p, *J* =7.8 Hz, 2H, -*CH_2_*-CH_2_S), 1.80 (p, *J* =7.2 Hz, 2H, -*CH_2_*-CH_2_I), 2.31 (s, 3H, -SCO*CH_3_*), 2.86 (t, *J* =7.5 Hz, 2H, -*CH_2_*-S), 3.17 (t, *J* =7.2 Hz, 2H, -*CH_2_*-I) ppm. ^13^C-NMR (75 MHz, CDCl_3_): δ =7.36 (-*C*-I), 28.59, 28.87, 29.14, 29.24, 29.41, 29.44, 29.59, 30.58, 30.75, 33.65, 196.09 (-S-*C*O-) ppm. EI-MS: 342 (M^+^).

We used the procedure reported in literature^5^ to synthesize 10-chloro-1-decanethioacetate. 1.00 g, 2.92 mmol 10-iodo-1-decanethioacetate was dissolved in 100 mL tetrahydrofuran (THF) in a two-neck flask, 3.20 g, 11.5 mmol tetrabutylammonium chloride (TBACl) was added to the flask and stirred at room temperature for 4 h, after which DI water was added to quench the reaction. Then, THF was removed under reduced pressure. The raw product was extract with DCM and washed with DI water for three times, dried over anhydrous Na_2_SO_4_, concentrated and followed by purification by column chromatography with hexane : ethyl acetate = 10 : 1. Yield 96 % (0.70g, 2.8 mmol). ^1^H-NMR (300 MHz, CDCl_3_): δ 1.28-1.41 (m, 12H, -(*CH_2_)_6_*-CH_2_CH_2_Cl), 1.56 (p, *J* =7.2 Hz, 2H, -*CH_2_*-CH_2_S), 1.76 (p, *J* =6.9 Hz, 2H, -*CH_2_*-CH_2_Cl), 2.32 (s, 3H, -SCO*CH_3_*), 2.86 (t, *J* =7.2 Hz, 2H, -*CH_2_*-S), 3.53 (t, *J* =6.9 Hz, 2H, -*CH_2_*-Cl) ppm. ^13^C-NMR (75 MHz, CDCl_3_): δ =27.01, 28.92, 28.99, 29.19, 29.29, 29.48, 29.49, 29.63, 30.77, 32.79, 45.30 (-*C*-Cl), 196.17 (-S-*C*) ppm. EI-MS: 250 (M^+^).

10-chloro-1-decanethiol was synthesized using the following method. To an oven-dried two-neck flask under N_2_, we poured 10-chloro-1-decanethioacetate (0.70 g, 2.8 mmol) and 20 mL methanol, and degassed for 20 min then cooled down with ice water. 3 mL acetyl chloride was added dropwise. After stirring at room temperature for 3 h, the reaction was quenched by DI water. DCM was used to extract the organic layer, the aqueous layer was extracted 3 times with DCM. Then, the organic layers were combined together and washed with DI water for three times. After anhydrous Na_2_SO_4_ was used to dry the organic layer and after filtration and concentration, the raw product was purified by column chromatography using hexane as eluent. Yield 81 % (0.48 g, 2.0 mmol). ^1^H-NMR (300 MHz, CDCl_3_): δ 1.32-1.42 (m, 12H, -*(CH_2_)_6_*-CH_2_CH_2_Cl), 1.63 (p, *J* =7.2 Hz, 2H, -*CH_2_*-CH_2_SH), 1.79 (p, *J* =6.9 Hz, 2H, -*CH_2_*-CH_2_Cl), 2.55 (q, *J* =7.2 Hz, 2H, -*CH_2_*-SH), 3.55 (t, *J* =6.9 Hz, 2H, -*CH_2_*-Cl) ppm. ^13^C-NMR (75 MHz, CDCl_3_): δ = 24.73 (-*C*-SH), 26.96, 28.44, 28.95, 29.12, 29.48, 32.74, 34.12, 45.24 (-*C*-Cl) ppm. EI-MS: 208 (M^+^). Melting point: 5.6 ℃.

**10-bromodecane-1-thiol** .10-bromodecane-1-thiol was synthesized following a procedure reported in literature.^6^ 10-bromodecene (2.00 g, 9.13 mmol), azobisisobutyronitrile (AIBN, 0.45 g, 2.7 mmol) was dissolved in 100 mL toluene in a two-neck flask. After 3 mL thioacetic acid was added to the mixture, the solution was allowed to reflux for 1 hour. The reaction mixture was cooled down to room temperature and washed with 100 mL saturated NaHCO_3_ aqueous solution. The aqueous layer was extracted with ether for 3 times. The combined organic layers was washed with DI water 3 times, separated, dried and concentrated. The product (2.10 g, 7.11 mmol) was found in the first band from the column with hexane : ethyl acetate = 10 :1 as eluent. Yield 77.9%. ^1^H-NMR (300 MHz, CDCl_3_): δ 1.28-1.41 (m, 12H, -*(CH_2_)_6_*-CH_2_ CH_2_Br), 1.55 (p, *J* = 7.2 Hz, 2H, -*CH_2_*-CH_2_-S), 1.84 (p, *J*= 6.9 Hz, 2H, -*CH_2_*-CH_2_-Br), 2.31 (s, 3H, -CH_2_-SCO*CH_3_*), 2.85 (t, *J* = 7.2 Hz, 2H, -*CH_2_*-SCOCH_3_), 3.39 (t, *J* = 6.9 Hz, 2H, -*CH_2_*-Br) ppm. ^13^C-NMR (75 MHz, CDCl_3_): δ = 28.27, 28.84, 28.89, 29.16, 29.26, 29.45, 29.61, 30.75, 32.95, 34.09, 196.10 (*C*=O) ppm. GC-MS: *m*/*z* 294 (M^+^).

10-bromo-1-decanethioacetate (1.00 g, 3.39 mmol) was dissolved in 10 mL methanol in an oven-dried two-neck flask, degassed for 15 min and then cooled down with ice water. 3 mL acetyl chloride was added dropwise, then the mixture was allowed to react for another 3 h. The methanol was removed in vacuo and the residue was dissolved with DCM and washed with DI water 3 times. The organic layer was dried over anhydrous MgSO_4_ and concentrated by rotary evaporator. The desired product was isolated from the first band by column chromatography with hexane. Yield 0.72 g, 2.8 mmol 10-bromo-1-decanethiol, 84 %. ^1^H-NMR (300 MHz, CDCl_3_): δ 1.28-1.39 (m, 12H, -*(CH_2_)_6_*-CH_2_CH_2_Br), 1.60 (p, *J* =7.2 Hz, 2H, -*CH_2_*-CH_2_SH), 1.84 (p, *J* =6.9 Hz, 2H, -*CH_2_*-CH_2_Br), 2.51 (q, *J* =7.2 Hz, 2H, -*CH_2_*-SH), 3.40 (t, *J* =6.9 Hz, 2H, -*CH_2_*-Br) ppm. ^13^C-NMR (75 MHz, CDCl_3_): δ = 24.75 (-*C*-SH), 28.27, 28.46, 28.84, 29.13, 29.46, 29.49, 32.94 (-*C*-Br), 34.13 ppm. EI-MS: 252 (M^+^). Melting point: 15.2 ℃.

**10-iododecane-1-thiol.** The synthetic procedure of 10-iododecane-1-thiol was taken from reference ^7^. We dissolved 10-iodo-1-decanethioacetate (1.20 g, 3.50 mmol) in 40 mL methanol and degassed the solution for 20 min. Subsequently, 6 mL 2 M aqueous HCl was added. The solution was refluxed for 5 h then cooled down to room temperature. The solvent was removed by rotary evaporator; DCM was used to extract the product. The organic layer was washed with DI water 3 times before dried over anhydrous Na_2_SO_4_. After filtration and concentration, the raw product was purified by column chromatography with hexane as eluent. Yield: 19 % (0.20 g, 6.7 mmol). ^1^H-NMR (300 MHz, CDCl_3_): δ 1.28-1.37 (m, 12H, -(*CH_2_)_6_*-CH_2_CH_2_I), 1.60 (p, *J* =7.2 Hz, 2H, -*CH_2_*-CH_2_SH), 1.81 (p, *J* =6.9 Hz, 2H, -*CH_2_*-CH_2_I), 2.51 (q, *J* =7.2 Hz, 2H, -*CH_2_*-SH), 3.18 (t, *J* =6.9 Hz, 2H, -*CH_2_*-I) ppm. ^13^C-NMR (75 MHz, CDCl_3_): δ =7.40 (-*C*-I), 24.77, 28.46, 28.62, 29.14, 29.45, 29.51, 30.60, 33.67, 34.14 ppm. EI-MS: 300 (M^+^). Melting point: 13.7 ℃.

**18-fluorooctadecane-1-thiol.** The synthesis of 18-fluorooctadecane-1-thiol was similar to that of 10-bromodecane-1-thiol. We first converted 18-bromo-octadecene to 18-fluoro-octadecene with tetrabutylammonium fluoride (TBAF) using a similar procedure reported in literature.^5^ 2.00 g (6.03 mmol) 18-bromo-octadecene and 24.1 (24.1 mmol, 4 eq.) mL TBAF (1 M in THF) were added into a two-neck flask under N_2_ then refluxed at room temperature for 4 h. THF was removed and diethy; ether was used to extract the organic product. The organic layers were combined and washed with DI water, dried, concentrated, and finally purified by column chromatography with hexane as the eluent. The yield of this step was 92.0 % (1.50 g, 5.55 mmol). ^1^H-NMR (400 MHz, CDCl_3_): δ 1.26-1.38 (m, 26H, -(*CH_2_)_13_*-CH_2_CH_2_F), 1.66-1.72 (m, 2H, -*CH_2_*-CH_2_-F), 2.04 (q, *J* = 5.1 Hz, 2H, -*CH_2_*-CH=CH_2_), 4.43 (dt, *J*_HF_ =47.2 Hz, *J*_HH_ =6.4 Hz, 2H, -*CH_2_*-F), 4.96 (tq, 2H,CH=C*H_2_*), 5.82 (m, 1H,C*H*=CH_2_)ppm. ^13^C-NMR (100 MHz, CDCl_3_): δ = 25.31 (d, ^3^*J*_CF_ = 5.3 Hz, -*C*-C-C-F), 29.12, 29.32, 29.41, 29.68, 29.71, 29.79, 29.30, 29.83, 30.58 (d, ^2^*J*_CF_ = 19.4 Hz, -*C*-C-F), 33.98, 84.35 (d, ^1^*J*_CF_ = 163.2 Hz, -*C*-F), 114.21(-C=*C*), 139.40 (-*C*=C) ppm. EI-MS: 269 (M^+^)

The 18-fluoro-octadecene was then converted to 18-fluoro-octadecanethioacetate using the previously mentioned method for 18-bromo-octadecanethioacetate.^6^18-fluoro-octadecene (1.40 g, 5.18 mmol) and azobisisobutyronitrile (AIBN, 0.28 g, 1.7 mmol) were dissolved in 100 mL toluene, thioacetic acid (3 mL, excess) was added subsequently. The mixture was refluxed for 1 h when TLC showed the reaction was finished. After cooling down to room temperature, 100 mL NaHCO_3_ aqueous solution was poured into the mixture to neutralize the excess thioacetic acid. The organic layer was separated and washed with DI water, brine, dried, and then concentrated. The product (1.50 g, 4.33 mmol, 83.6 % yield) was isolated from the first band from the column with ethyl acetate : hexane = 1 : 10 as the eluent. ^1^H-NMR (400 MHz, CDCl_3_): δ 1.25-1.36 (m, 28H, -(*CH_2_)_14_*-CH_2_CH_2_I), 1.56-1.72 (m, 4H, -*CH_2_*-CH_2_S and -*CH_2_*-CH_2_F), 2.32 (s, 3H, -SCO*CH_3_*), 2.86 (t, *J* =7.2 Hz, 2H, -*CH_2_*-S), 4.43 (dt, *J*_HF_ =47.6 Hz, *J*_HH_ =6.4 Hz, 2H, -*CH_2_*-F) ppm. ^13^C-NMR (100 MHz, CDCl_3_): δ = 25.29 (d, ^3^*J*_CF_ = 5.7 Hz, -*C*-C-C-F), 28.98, 29.27, 29.32, 29.39, 29.62, 29.65, 29.70, 29.72, 29.78, 29.81, 30.57, (d, ^2^*J*_CF_ = 19.1 Hz, -*C*-C-F), 30.76, 83.88 (d, ^1^*J*_CF_ = 163.1 Hz, -*C*-F), 196.17 (-*C*=O) ppm. EI-MS: 346 (M^+^).

18-fluorooctadecane-1-thiol was synthesized according to the following method. 0.50 g (1.4 mmol) 18-fluoro-octadecanethioacetate was first dissolved in 20 mL DCM, 30 mL methanol was added subsequently. 1.5 mL excess acetyl chloride was added dropwise after the solution was cooled with by ice water. After the mixture was allowed to react for 3 h, the organic solvents were removed under reduced pressure. The residue was picked up by DCM and washed with DI water for 3 times, separated, dried, concentrated, and purified with column chromatography with hexane as the eluent. This step yielded 1.40 g (1.31 mmol) product with yield of 93 %. ^1^H-NMR (400 MHz, CDCl_3_): δ 1.25-1.37 (m, 28H, -*(CH_2_)_14_*-CH_2_CH_2_F), 1.60-1.71 (m, 4H, -*CH_2_*-CH_2_F and -*CH_2_*-CH_2_SH), 2.51 (q, *J* =7.2 Hz, 2H, -*CH_2_*-SH), 4.43 (dt, *J*_HF_ =47.2 Hz, *J*_HH_ =6.4 Hz, 2H, -*CH_2_*-F) ppm. ^13^C-NMR (100 MHz, CDCl_3_): δ = 24.76, 25.29 (d, ^3^*J*_CF_ = 5.5 Hz, -*C*-C-C-F), 28.52, 29.22, 29.38, 29.66, 29.69, 29.73, 29.78, 29.80, 30.56 (d, ^2^*J*_CF_ = 19.2 Hz, -*C*-C-F), 34.19, 84.29 (d, ^1^*J*_CF_ = 163.2 Hz, -*C*-F) ppm. EI-MS: 303 (M^+^). Melting point: 32.3 ℃.

16-bromo-1-hexadecanethioacetate was synthesized from 16-bromohexadecene as follows.^6, 8^ 4.00 g (168 mmol) magnesium turnings were added to 100 mL distilled diethyl ether followed by addition of 6.70 g (49.6 mmol) 4-bromo-1-butene. The mixture was refluxed for 6 h. 32.80 g (99.96 mmol) 1,12-dibromododecane was dissolved in 100 mL distilled THF and cooled down by ice water. The previously prepared Grignard reagent was rapidly transferred dropwise into the THF mixture, followed by 1.00 mL dilithium tetrachlorocuprate(II) solution (0.1 M Li_2_CuCl_4_ in THF). The mixture was warmed to room temperature and reacted for 16 h, hydrolyzed with 100 mL saturated aqueous NH_4_Cl solution. The organic phase was separated; the aqueous phase was extracted 3 times with diethyl ether. We combined the organic extracts followed by washing 3 times with DI water, separated, dried over N_2_SO_4_, and concentrated the organic layer. The raw product was used for the next step without further purification. We dissolved the raw product in 100 mL toluene, added 8 mL thioacetic acid to the solution and refluxed for 1 h. 100 mL NaHCO_3_ aqueous solution was added after the solution was cooled to room temperature. The organic layer was separated; the aqueous layer was extracted with diethyl ether for 3 times. The combined organic extracts was then washed with DI water 3 times, separated, dried, and concentrated. The 16-bromo-1-hexadecanethioacetate was isolated form the second band from the column with hexane and ethyl acetate (10 : 1) as eluent. Yield: 18.8 % (3.80 g, 9.32 mmol) 16-bromo-1-hexadecanethioacetate. ^1^H-NMR (400 MHz, CDCl_3_): δ 1.25-1.42 (m, 24H, -*(CH_2_)_12_*-CH_2_ CH_2_Br), 1.56 (p, *J* = 7.2 Hz, 2H, -*CH_2_*-CH_2_-S), 1.85 (p, *J* = 6.8 Hz, 2H, -*CH_2_*-CH_2_-Br), 2.31 (s, 3H, -CH_2_-SCO*CH_3_*), 2.86 (t, *J* = 7.2 Hz, 2H, -*CH_2_*-SCOCH_3_), 3.40 (t, *J* = 6.8 Hz, 2H, -*CH_2_*-Br) ppm. ^13^C-NMR (100 MHz, CDCl_3_): δ = 28.33, 28.91, 28.97, 29.26, 29.31, 29.58, 29.61, 29.64, 29.68, 29.70, 29.75, 29.77, 30.76, 33.00, 34.14, 196.15 (*C*=O) ppm. EI-MS: 378 (M^+^).

16-iodo-1-hexadecanethioacetate and 18-iodo-1-octadecanethioacetate were synthesized as follows. 16-bromo-1-hexadecanethioacetate (3.50 g, 9.22 mmol) and sodium iodide were added into 150 mL acetone, refluxed for 5 h, cooled down, followed by removal of the acetone under reduced pressure. The residue was dissolved in diethyl ether, filtered, and washed with DI water. The organic layer was dried over Na_2_SO_4_, filtered and concentrated after which the raw product was purified with column chromatography with ethyl acetate and hexane (1: 10). Yield: 3.50 g (8.21 mmol) 16-iodo-1-hexadecanethioacetate (89.0 % yield). ^1^H-NMR (300 MHz, CDCl_3_): δ 1.25-1.36 (m, 24H, -(*CH_2_)_12_*-CH_2_CH_2_I), 1.56 (p, *J* =7.6 Hz, 2H, -*CH_2_*-CH_2_S), 1.82 (p, *J* =7.2 Hz, 2H, -*CH_2_*-CH_2_I), 2.32 (s, 3H, -SCO*CH_3_*), 2.86 (t, *J* =7.2 Hz, 2H, -*CH_2_*-S), 3.19 (t, *J* =7.2 Hz, 2H, -*CH_2_*-I) ppm. ^13^C-NMR (75 MHz, CDCl_3_): δ =7.42 (-*C*-I), 28.70, 28.98, 29.27, 29.33, 29.57, 29.62, 29.65, 29.69, 29.72, 29.76, 29.77, 29.78, 30.67, 30.78, 33.75, 196.18 (-S-*C*O-) ppm. EI-MS: 426 (M^+^).

18-iodo-1-octadecaneathiocetate: ^1^H-NMR (400 MHz, CDCl_3_): δ 1.25-1.37 (m, 26H, -(*CH_2_)_13_*-CH_2_CH_2_I), 1.56 (p, *J* =7.5 Hz, 2H, -*CH_2_*-CH_2_S), 1.82 (p, *J* =6.9 Hz, 2H, -*CH_2_*-CH_2_I), 2.32 (s, 3H, -SCO*CH_3_*), 2.86 (t, *J* =7.5 Hz, 2H, -*CH_2_*-S), 3.18 (t, *J* =6.9 Hz, 2H, -*CH_2_*-I) ppm. ^13^C-NMR (100 MHz, CDCl_3_): δ =7.39 (-*C*-I), 28.70, 28.98, 29.27, 29.32, 29.57, 29.63, 29.65, 29.69, 29.72, 29.76, 29.77, 29.81, 30.67, 30.77, 33.74, 196.16 (-S-*C*O-) ppm. EI-MS: 454 (M^+^).

1-bromo-12-fluorododecane. ^1^H-NMR (400 MHz, CDCl_3_): δ 1.28-1.40 (m, 16H, -*(CH_2_)_8_*-CH_2_CH_2_F), 1.65-1.71(m, 2H, -*CH_2_*-CH_2_F), 1.85 (p, *J* =6.8 Hz, 2H, -*CH_2_*-CH_2_Br), 3.40 (t, *J* =6.8 Hz, 2H, -*CH_2_*-Br), 4.42 (dt, *J*_HF_ =47.6 Hz, *J*_HH_ =6.4 Hz, 2H, -*CH_2_*-F) ppm. ^13^C-NMR (100 MHz, CDCl_3_): δ = 25.28 (d, ^3^*J*_CF_ = 5.3 Hz, -*C*-C-C-F), 28.30, 28.89, 29.36, 29.54, 29.61, 30.55 (d, ^2^*J*_CF_ = 19.1 Hz, -*C*-C-F), 32.98, 34.08, 84.30 (d, ^1^*J*_CF_ = 163.0 Hz, -*C*-F) ppm. EI-MS: 268 (M^+^).

1-bromo-14-fluorotetradecane. ^1^H-NMR (400 MHz, CDCl_3_): δ 1.27-1.40 (m, 20H, -*(CH_2_)_10_*-CH_2_CH_2_F), 1.65-1.72(m, 2H, -*CH_2_*-CH_2_F), 1.85 (p, *J* =6.8 Hz, 2H, -*CH_2_*-CH_2_Br), 3.40 (t, *J* =6.8 Hz, 2H, -*CH_2_*-Br), 4.43 (dt, *J*_HF_ =47.6 Hz, *J*_HH_ =6.4 Hz, 2H, -*CH_2_*-F) ppm. ^13^C-NMR (100 MHz, CDCl_3_): δ = 25.29 (d, ^3^*J*_CF_ = 5.6 Hz, -*C*-C-C-F), 28.32, 28.91, 29.38, 29.57, 29.65, 29.67, 29.73, 30.56 (d, ^2^*J*_CF_ = 19.3 Hz, -*C*-C-F), 32.99, 34.12, 84.35 (d, ^1^*J*_CF_ = 162.9 Hz, -*C*-F) ppm. EI-MS: 296 (M^+^).

1-bromo-16-fluorohexadecane. ^1^H-NMR (400 MHz, CDCl_3_): δ 1.26-1.40 (m, 24H, -*(CH_2_)_12_*-CH_2_CH_2_F), 1.66-1.72(m, 2H, -*CH_2_*-CH_2_F), 1.85 (p, *J* =6.8 Hz, 2H, -*CH_2_*-CH_2_Br), 3.40 (t, *J* =6.8 Hz, 2H, -*CH_2_*-Br), 4.43 (dt, *J*_HF_ =47.6 Hz, *J*_HH_ =6.4 Hz, 2H, -*CH_2_*-F) ppm. ^13^C-NMR (100 MHz, CDCl_3_): δ = 25.30 (d, ^3^*J*_CF_ = 5.6 Hz, -*C*-C-C-F), 28.33, 28.92, 29.39, 29.59, 29.66, 29.69, 29.76, 29.78, 30.57 (d, ^2^*J*_CF_ = 19.3 Hz, -*C*-C-F), 33.01, 34.14, 84.37 (d, ^1^*J*_CF_ = 163.2 Hz, -*C*-F) ppm. EI-MS: 324 (M^+^).

1-fluoro-12-thiocyanatododecane. ^1^H-NMR (400 MHz, CDCl_3_): δ 1.27-1.42 (m, 16H, -*(CH_2_)_8_*-CH_2_CH_2_F), 1.64-1.70 (m, 2H, -*CH_2_*-CH_2_F), 1.81 (p, *J* =7.2 Hz, 2H, -*CH_2_*-CH_2_SCN), 2.93 (t, *J* =7.2 Hz, 2H, -*CH_2_*-SCN), 4.42 (dt, *J*_HF_ =47.6 Hz, *J*_HH_ =6.4 Hz, 2H, -*CH_2_*-F) ppm. ^13^C-NMR (100 MHz, CDCl_3_): δ = 25.22 (d, ^3^*J*_CF_ = 5.6 Hz, -*C*-C-C-F), 28.01, 28.93, 29.28, 29.39, 29.51, 29.53, 29.95, 30.48 (d, ^2^*J*_CF_ = 19.2 Hz, -*C*-C-F), 34.15, 84.24 (d, ^1^*J*_CF_ = 163.1 Hz, -*C*-F), 112.43 (-S-*C*≡N)ppm. EI-MS: 244 (M^+^).

1-fluoro-14-thiocyanatotetradecane. ^1^H-NMR (400 MHz, CDCl_3_): δ 1.26-1.43 (m, 20H, -*(CH_2_)_10_*-CH_2_CH_2_F), 1.65-1.71 (m, 2H, -*CH_2_*-CH_2_F), 1.81 (p, *J* =7.2 Hz, 2H, -*CH_2_*-CH_2_SCN), 2.94 (t, *J* =7.2 Hz, 2H, -*CH_2_*-SCN), 4.42 (dt, *J*_HF_ =47.6 Hz, *J*_HH_ =6.4 Hz, 2H, -*CH_2_*-F) ppm. ^13^C-NMR (100 MHz, CDCl_3_): δ = 25.25 (d, ^3^*J*_CF_ = 5.5 Hz, -*C*-C-C-F), 28.06, 28.97, 29.34, 29.45, 29.58, 29.60, 29.62, 29.65, 29.68, 29.99, 30.52 (d, ^2^*J*_CF_ = 19.4 Hz, -*C*-C-F), 34.19, 84.31 (d, ^1^*J*_CF_ = 163.1 Hz, -*C*-F), 112.47 (-S-*C*≡N) ppm. EI-MS: 272 (M^+^).

1-fluoro-16-thiocyanatohexadecane. ^1^H-NMR (400 MHz, CDCl_3_): δ 1.26-1.43 (m, 24H, -*(CH_2_)_12_*-CH_2_CH_2_F), 1.65-1.72 (m, 2H, -*CH_2_*-CH_2_F), 1.80 (p, *J* =7.2 Hz, 2H, -*CH_2_*-CH_2_SCN), 2.94 (t, *J* =7.2 Hz, 2H, -*CH_2_*-SCN), 4.43 (dt, *J*_HF_ =47.6 Hz, *J*_HH_ =6.4 Hz, 2H, -*CH_2_*-F) ppm. ^13^C-NMR (100 MHz, CDCl_3_): δ = 25.29 (d, ^3^*J*_CF_ = 5.6 Hz, -*C*-C-C-F), 28.12, 29.02, 29.39, 29.50, 29.64, 29.65, 29.68, 29.72, 29.76, 30.03, 30.56 (d, ^2^*J*_CF_ = 19.1 Hz, -*C*-C-F), 34.25, 84.38 (d, ^1^*J*_CF_ = 163.0 Hz, -*C*-F), 112.52 (-S-*C*≡N) ppm. EI-MS: 301 (M^+^).

12-fluorododecane-1-thiol. ^1^H-NMR (400 MHz, CDCl_3_): δ 1.27-1.39 (m, 16H, -*(CH_2_)_8_*-CH_2_CH_2_F), 1.61-1.72 (m, 4H, -*CH_2_*-CH_2_F and -*CH_2_*-CH_2_SH), 2.52 (q, *J* =7.2 Hz, 2H, -*CH_2_*-SH), 4.44 (dt, *J*_HF_ =47.6 Hz, *J*_HH_ =6.4 Hz, 2H, -*CH_2_*-F) ppm. ^13^C-NMR (75 MHz, CDCl_3_): δ = 24.77, 25.29 (d, ^3^*J*_CF_ = 5.5 Hz, -*C*-C-C-F), 28.51, 29.20, 29.37, 29.63, 29.66, 30.56 (d, ^2^*J*_CF_ = 19.2 Hz, -*C*-C-F), 34.18, 84.32 (d, ^1^*J*_CF_ = 163.0 Hz, -*C*-F) ppm. EI-MS: 220 (M^+^). Melting point: -1.8 ℃.

14-fluorotetradecane-1-thiol. ^1^H-NMR (400 MHz, CDCl_3_): δ 1.26-1.39 (m, 20H, -*(CH_2_)_10_*-CH_2_CH_2_F), 1.61-1.72 (m, 4H, -*CH_2_*-CH_2_F and -*CH_2_*-CH_2_SH), 2.52 (q, *J* =7.2 Hz, 2H, -*CH_2_*-SH), 4.43 (dt, *J*_HF_ =47.6 Hz, *J*_HH_ =6.4 Hz, 2H, -*CH_2_*-F) ppm. ^13^C-NMR (75 MHz, CDCl_3_): δ = 24.76, 25.29 (d, ^3^*J*_CF_ = 5.5 Hz, -*C*-C-C-F), 28.51, 29.21, 29.38, 29.65, 29.70, 29.74, 30.56 (d, ^2^*J*_CF_ = 19.2 Hz, -*C*-C-F), 34.19, 84.30 (d, ^1^*J*_CF_ = 163.0 Hz, -*C*-F) ppm. EI-MS: 248 (M^+^). Melting point: 12.1 ℃.

16-fluorohexadecane-1-thiol. ^1^H-NMR (300 MHz, CDCl_3_): δ 1.26-1.39 (m, 24H, -*(CH_2_)_12_*-CH_2_CH_2_F), 1.60-1.72 (m, 4H, -*CH_2_*-CH_2_F and -*CH_2_*-CH_2_SH), 2.52 (q, *J* =7.2 Hz, 2H, -*CH_2_*-SH), 4.43 (dt, *J*_HF_ =47.6 Hz, *J*_HH_ =6.4 Hz, 2H, -*CH_2_*-F) ppm. ^13^C-NMR (100 MHz, CDCl_3_): δ = 24.77, 25.30 (d, ^3^*J*_CF_ = 5.5 Hz, -*C*-C-C-F), 28.53, 29.23, 29.39, 29.66, 29.69, 29.73, 29.79, 30.57 (d, ^2^*J*_CF_ = 19.2 Hz, -*C*-C-F), 34.21, 84.34 (d, ^1^*J*_CF_ = 163.1 Hz, -*C*-F) ppm. EI-MS: 303 (M^+^). Melting point: 24.0 ℃.

1,14-diiodotetradecane. ^1^H-NMR (300 MHz, CDCl_3_): δ 1.25-1.37 (m, 20H, -(*CH_2_)_10_*-CH_2_CH_2_I), 1.80 (p, *J* =7.2 Hz, 4H, -*CH_2_*-CH_2_I), 3.17 (t, *J* =7.2 Hz, 4H, -*CH_2_*-I) ppm. ^13^C-NMR (75 MHz, CDCl_3_): δ =7.35 (-*C*-I), 28.65, 29.52, 29.63, 29.68, 30.62, 33.69 ppm. EI-MS: *m*/*z* 450 (M^+^).

12-iodo-1-dodecanethioacetate. ^1^H-NMR (300 MHz, CDCl_3_): δ 1.25-1.36 (m, 16H, -(*CH_2_)_8_*-CH_2_CH_2_I), 1.55 (p, *J* =7.2 Hz, 2H, -*CH_2_*-CH_2_S), 1.81 (p, *J* =7.2 Hz, 2H, -*CH_2_*-CH_2_I), 2.31 (s, 3H, -SCO*CH_3_*), 2.85 (t, *J* =7.2 Hz, 2H, -*CH_2_*-S), 3.17 (t, *J* =7.2 Hz, 2H, -*CH_2_*-I) ppm. ^13^C-NMR (75 MHz, CDCl_3_): δ =7.39 (-*C*-I), 28.64, 28.92, 29.20, 29.27, 29.50, 29.54, 29.60, 30.61, 30.76, 33.68, 196.09 (-S-*C*O-) ppm. EI-MS: *m*/*z* 369.9 (M^+^).

14-iodo-1-tetradecanethioacetate. ^1^H-NMR (300 MHz, CDCl_3_): δ 1.24-1.36 (m, 20H, -(*CH_2_)_10_*-CH_2_CH_2_I), 1.55 (p, *J* =7.2 Hz, 2H, -*CH_2_*-CH_2_S), 1.80 (p, *J* =7.2 Hz, 2H, -*CH_2_*-CH_2_I), 2.30 (s, 3H, -SCO*CH_3_*), 2.84 (t, *J* =7.2 Hz, 2H, -*CH_2_*-S), 3.17 (t, *J* =7.2 Hz, 2H, -*CH_2_*-I) ppm. ^13^C-NMR (75 MHz, CDCl_3_): δ =7.36 (-*C*-I), 28.64, 28.92, 29.21, 29.26, 29.51, 29.56, 29.61,29.63, 29.65, 29.68, 30.61, 30.74, 33.69, 196.05 (-S-*C*O-) ppm. EI-MS: *m*/*z* 398 (M^+^).

12-chloro-1-dodecanethioacetate. ^1^H-NMR (300 MHz, CDCl_3_): δ 1.26-1.42 (m, 16H, -(*CH_2_)_8_*-CH_2_CH_2_Cl), 1.55 (p, *J* =7.2 Hz, 2H, -*CH_2_*-CH_2_S), 1.76 (p, *J* =6.9 Hz, 2H, -*CH_2_*-CH_2_Cl), 2.31 (s, 3H, -SCO*CH_3_*), 2.85 (t, *J* =7.2 Hz, 2H, -*CH_2_*-S), 3.52 (t, *J* =6.9 Hz, 2H, -*CH_2_*-Cl) ppm. ^13^C-NMR (75 MHz, CDCl_3_): δ =27.01, 28.93, 29.00, 29.22, 29.27, 29.56, 29.62, 30.74, 32.78, 45.28 (-*C*-Cl), 196.12 (-S-*C*) ppm. EI-MS: *m*/*z* 278.1 (M^+^).

14- chloro-1-tetradecanethioacetate. ^1^H-NMR (300 MHz, CDCl_3_): δ 1.25-1.41 (m, 20H, -(*CH_2_)_10_*-CH_2_CH_2_Cl), 1.55 (p, *J* =7.2 Hz, 2H, -*CH_2_*-CH_2_S), 1.76 (p, *J* =6.9 Hz, 2H, -*CH_2_*-CH_2_Cl), 2.31 (s, 3H, -SCO*CH_3_*), 2.85 (t, *J* =7.2 Hz, 2H, -*CH_2_*-S), 3.52 (t, *J* =6.9 Hz, 2H, -*CH_2_*-Cl) ppm. ^13^C-NMR (75 MHz, CDCl_3_): δ =27.02, 28.95, 29.02, 29.23, 29.28, 29.58, 29.63, 29.65, 29.67, 29.71, 30.74, 32.79, 45.27 (-*C*-Cl), 196.09 (-S-*C*) ppm. EI-MS: *m*/*z* 306.1 (M^+^).

16- chloro-1-hexadecanethioacetate. ^1^H-NMR (400 MHz, CDCl_3_): δ 1.24-1.41 (m, 24H, -(*CH_2_)_12_*-CH_2_CH_2_Cl), 1.55 (p, *J* =7.2 Hz, 2H, -*CH_2_*-CH_2_S), 1.75 (p, *J* =6.9 Hz, 2H, -*CH_2_*-CH_2_Cl), 2.31 (s, 3H, -SCO*CH_3_*), 2.85 (t, *J* =7.2 Hz, 2H, -*CH_2_*-S), 3.51 (t, *J* =6.9 Hz, 2H, -*CH_2_*-Cl) ppm. ^13^C-NMR (100 MHz, CDCl_3_): δ =27.02, 28.95, 29.02, 29.24, 29.28, 29.59, 29.63, 29.67, 29.69, 29.74, 20.76, 30.73, 32.79, 45.25 (-*C*-Cl), 196.06 (-S-*C*) ppm. EI-MS: *m*/*z* 334.1 (M^+^).

18- chloro-1-octadecanethioacetate. ^1^H-NMR (300 MHz, CDCl_3_): δ 1.24-1.41 (m, 28H, -(*CH_2_)_14_*-CH_2_CH_2_Cl), 1.56 (p, *J* =7.2 Hz, 2H, -*CH_2_*-CH_2_S), 1.76 (p, *J* =6.9 Hz, 2H, -*CH_2_*-CH_2_Cl), 2.31 (s, 3H, -SCO*CH_3_*), 2.86 (t, *J* =7.2 Hz, 2H, -*CH_2_*-S), 3.52 (t, *J* =6.9 Hz, 2H, -*CH_2_*-Cl) ppm. ^13^C-NMR (75 MHz, CDCl_3_): δ =27.05, 28.97, 29.04, 29.26, 29.32, 29.62, 29.65, 29.69, 29.72, 29.77, 29.80, 30.75, 32.82, 45.29 (-*C*-Cl), 196.13 (-S-*C*) ppm. EI-MS: *m*/*z* 362.1 (M^+^).

12-chloro-1-dodecanethiol. ^1^H-NMR (300 MHz, CDCl_3_): δ 1.26-1.38 (m, 16H, -*(CH_2_)_8_*-CH_2_CH_2_Cl), 1.59 (p, *J* =7.2 Hz, 2H, -*CH_2_*-CH_2_SH), 1.75 (p, *J* =6.9 Hz, 2H, -*CH_2_*-CH_2_Cl), 2.51 (q, *J* =7.2 Hz, 2H, -*CH_2_*-SH), 3.51 (t, *J* =6.9 Hz, 2H, -*CH_2_*-Cl) ppm. ^13^C-NMR (75 MHz, CDCl_3_): δ = 24.73 (-*C*-SH), 26.97, 28.46, 28.97, 29.15, 29.53, 29.58, 29.61, 32.75, 34.14, 45.22 (-*C*-Cl) ppm. EI-MS: 236 (M^+^). Melting point: 21.5 ℃.

14-chloro-1-tetradecanethiol. ^1^H-NMR (300 MHz, CDCl_3_): δ 1.26-1.39 (m, 20H, -*(CH_2_)_10_*-CH_2_CH_2_Cl), 1.60 (p, *J* =7.2 Hz, 2H, -*CH_2_*-CH_2_SH), 1.76 (p, *J* =6.9 Hz, 2H, -*CH_2_*-CH_2_Cl), 2.51 (q, *J* =7.2 Hz, 2H, -*CH_2_*-SH), 3.52 (t, *J* =6.9 Hz, 2H, -*CH_2_*-Cl) ppm. ^13^C-NMR (75 MHz, CDCl_3_): δ = 24.76 (-*C*-SH), 27.01, 28.50, 29.01, 29.19, 29.57, 29.64, 29.69, 29.71, 32.78, 34.17, 45.26 (-*C*-Cl) ppm. EI-MS: 264 (M^+^). Melting point: 24.0 ℃.

16-chloro-1-hexadecanethiol. ^1^H-NMR (400 MHz, CDCl_3_): δ 1.25-1.39 (m, 24H, -*(CH_2_)_12_*-CH_2_CH_2_Cl), 1.59 (p, *J* =7.2 Hz, 2H, -*CH_2_*-CH_2_SH), 1.75 (p, *J* =6.9 Hz, 2H, -*CH_2_*-CH_2_Cl), 2.50 (q, *J* =7.2 Hz, 2H, -*CH_2_*-SH), 3.51 (t, *J* =6.9 Hz, 2H, -*CH_2_*-Cl) ppm. ^13^C-NMR (100 MHz, CDCl_3_): δ = 24.78 (-*C*-SH), 27.03, 28.52, 29.03, 29.21, 29.60, 29.66, 29.68, 29.72, 29.75, 29.77, 32.80, 34.19, 45.29 (-*C*-Cl) ppm. EI-MS: 292 (M^+^). Melting point: 40.2 ℃.

18-chloro-1-octadecanethiol. ^1^H-NMR (300 MHz, CDCl_3_): δ 1.25-1.39 (m, 28H, -*(CH_2_)_14_*-CH_2_CH_2_Cl), 1.60 (p, *J* =7.2 Hz, 2H, -*CH_2_*-CH_2_SH), 1.76 (p, *J* =6.9 Hz, 2H, -*CH_2_*-CH_2_Cl), 2.52 (q, *J* =7.2 Hz, 2H, -*CH_2_*-SH), 3.53 (t, *J* =6.9 Hz, 2H, -*CH_2_*-Cl) ppm. ^13^C-NMR (75 MHz, CDCl_3_): δ = 24.80 (-*C*-SH), 27.05, 28.54, 29.05, 29.23, 29.62, 29.67, 29.70, 29.74, 29.81, 32.82, 34.21, 45.32 (-*C*-Cl) ppm. EI-MS: 320 (M^+^). Melting point: 49.2 ℃.

12-bromo-1-dodecanethioacetate. ^1^H-NMR (300 MHz, CDCl_3_): δ 1.26-1.42 (m, 16H, -*(CH_2_)_8_*-CH_2_ CH_2_Br), 1.56 (p, *J* = 7.2 Hz, 2H, -*CH_2_*-CH_2_-S), 1.85 (p, *J*= 6.9 Hz, 2H, -*CH_2_*-CH_2_-Br), 2.32 (s, 3H, -CH_2_-SCO*CH_3_*), 2.86 (t, *J* = 7.2 Hz, 2H, -*CH_2_*-SCOCH_3_), 3.40 (t, *J* = 6.9 Hz, 2H, -*CH_2_*-Br) ppm. ^13^C-NMR (75 MHz, CDCl_3_): δ = 28.31, 28.89, 28.95, 29.23, 29.30, 29.55, 29.57, 29.63, 30.77, 32.98, 34.15, 196.17 (*C*=O) ppm. EI-MS: 322 (M^+^).

14- bromo-1-tetradecanethioacetate. ^1^H-NMR (300 MHz, CDCl_3_): δ 1.26-1.42 (m, 20H, -*(CH_2_)_10_*-CH_2_ CH_2_Br), 1.56 (p, *J* = 7.2 Hz, 2H, -*CH_2_*-CH_2_-S), 1.85 (p, *J*= 6.9 Hz, 2H, -*CH_2_*-CH_2_-Br), 2.32 (s, 3H, -CH_2_-SCO*CH_3_*), 2.86 (t, *J* = 7.2 Hz, 2H, -*CH_2_*-SCOCH_3_), 3.40 (t, *J* = 6.9 Hz, 2H, -*CH_2_*-Br) ppm. ^13^C-NMR (75 MHz, CDCl_3_): δ = 28.31, 28.89, 28.95, 29.23, 29.30, 29.55, 29.57, 29.63, 30.77, 32.98, 34.15, 196.17 (*C*=O) ppm. EI-MS: 352 (M^+^).

18-bromo-1-octadecanethioacetate. ^1^H-NMR (300 MHz, CDCl_3_): δ 1.25-1.41 (m, 28H, -*(CH_2_)_14_*-CH_2_ CH_2_Br), 1.56 (p, *J* = 7.2 Hz, 2H, -*CH_2_*-CH_2_-S), 1.85 (p, *J*= 6.9 Hz, 2H, -*CH_2_*-CH_2_-Br), 2.32 (s, 3H, -CH_2_-SCO*CH_3_*), 2.86 (t, *J* = 7.2 Hz, 2H, -*CH_2_*-SCOCH_3_), 3.40 (t, *J* = 6.9 Hz, 2H, -*CH_2_*-Br) ppm. ^13^C-NMR (75 MHz, CDCl_3_): δ = 28.34, 28.93, 28.98, 29.27, 29.33, 29.59, 29.63, 29.66, 29.69, 29.73, 29.78, 29.81, 30.78, 33.01, 34.16, 196.18 (*C*=O) ppm. EI-MS: 408.2 (M^+^).

12-bromo-1-dodecanethiol. ^1^H-NMR (300 MHz, CDCl_3_): δ 1.27-1.39 (m, 16H, -*(CH_2_)_8_*-CH_2_CH_2_Br), 1.61 (p, *J* =7.2 Hz, 2H, -*CH_2_*-CH_2_SH), 1.85 (p, *J* =6.9 Hz, 2H, -*CH_2_*-CH_2_Br), 2.52 (q, *J* =7.2 Hz, 2H, -*CH_2_*-SH), 3.40 (t, *J* =6.9 Hz, 2H, -*CH_2_*-Br) ppm. ^13^C-NMR (75 MHz, CDCl_3_): δ = 24.79 (-*C*-SH), 28.32, 28.51, 28.90, 29.20, 29.55, 29.63, 29.65, 32.99 (-*C*-Br), 34.14, 34.18 ppm. EI-MS: 280 (M^+^). Melting point: 28.8 ℃.

14-bromo-1-tetradecanethiol. ^1^H-NMR (300 MHz, CDCl_3_): δ 1.26-1.39 (m, 20H, -*(CH_2_)_10_*-CH_2_CH_2_Br), 1.60 (p, *J* =7.2 Hz, 2H, -*CH_2_*-CH_2_SH), 1.85 (p, *J* =6.9 Hz, 2H, -*CH_2_*-CH_2_Br), 2.51 (q, *J* =7.2 Hz, 2H, -*CH_2_*-SH), 3.40 (t, *J* =6.9 Hz, 2H, -*CH_2_*-Br) ppm. ^13^C-NMR (75 MHz, CDCl_3_): δ = 24.78 (-*C*-SH), 28.31, 28.51, 28.90, 29.20, 29.56, 29.64, 29.65, 29.70, 29.72, 32.98 (-*C*-Br), 34.14, 34.18 ppm. EI-MS: 310 (M^+^). Melting point: 39.8 ℃.

16-bromo-1-hexadecanethiol. ^1^H-NMR (400 MHz, CDCl_3_): δ 1.25-1.39 (m, 24H, -*(CH_2_)_12_*-CH_2_CH_2_Br), 1.60 (p, *J* =7.2 Hz, 2H, -*CH_2_*-CH_2_SH), 1.84 (p, *J* =6.9 Hz, 2H, -*CH_2_*-CH_2_Br), 2.51 (q, *J* =7.2 Hz, 2H, -*CH_2_*-SH), 3.39 (t, *J* =6.9 Hz, 2H, -*CH_2_*-Br) ppm. ^13^C-NMR (100 MHz, CDCl_3_): δ = 24.77 (-*C*-SH), 28.31, 28.51, 28.90, 29.21, 29.56, 29.65, 29.66, 29.71, 29.73, 29.76, 32.98 (-*C*-Br), 34.09, 34.18 ppm. EI-MS: 336 (M^+^). Melting point: 45.4 ℃.

18-bromo-1-octadecanethiol. ^1^H-NMR (300 MHz, CDCl_3_): δ 1.25-1.39 (m, 28H, -*(CH_2_)_14_*-CH_2_CH_2_Br), 1.60 (p, *J* =7.2 Hz, 2H, -*CH_2_*-CH_2_SH), 1.85 (p, *J* =6.9 Hz, 2H, -*CH_2_*-CH_2_Br), 2.52 (q, *J* =7.2 Hz, 2H, -*CH_2_*-SH), 3.40 (t, *J* =6.9 Hz, 2H, -*CH_2_*-Br) ppm. ^13^C-NMR (75 MHz, CDCl_3_): δ = 24.80 (-*C*-SH), 28.33, 28.53, 28.92, 29.23, 29.59, 29.67, 29.69, 29.74, 29.76, 29.81, 33.00 (-*C*-Br), 34.16, 34.20 ppm. EI-MS: 366 (M^+^). Melting point: 54.4 ℃.

12-iodo-1-dodecanethiol. ^1^H-NMR (300 MHz, CDCl_3_): δ 1.27-1.37 (m, 16H, -(*CH_2_)_8_*-CH_2_CH_2_I), 1.60 (p, *J* =7.2 Hz, 2H, -*CH_2_*-CH_2_SH), 1.81 (p, *J* =6.9 Hz, 2H, -*CH_2_*-CH_2_I), 2.51 (q, *J* =7.2 Hz, 2H, -*CH_2_*-SH), 3.18 (t, *J* =6.9 Hz, 2H, -*CH_2_*-I) ppm. ^13^C-NMR (75 MHz, CDCl_3_): δ =7.40 (-*C*-I), 24.79, 28.51, 28.67, 29.20, 29.53, 29.62, 30.64, 33.71, 34.18 ppm. EI-MS: 328 (M^+^). Melting point: 28.2 ℃.

14-iodo-1-tetradecanethiol. ^1^H-NMR (300 MHz, CDCl_3_): δ 1.26-1.37 (m, 20H, -(*CH_2_)_10_*-CH_2_CH_2_I), 1.60 (p, *J* =7.2 Hz, 2H, -*CH_2_*-CH_2_SH), 1.82 (p, *J* =6.9 Hz, 2H, -*CH_2_*-CH_2_I), 2.52 (q, *J* =7.2 Hz, 2H, -*CH_2_*-SH), 3.18 (t, *J* =6.9 Hz, 2H, -*CH_2_*-I) ppm. ^13^C-NMR (75 MHz, CDCl_3_): δ =7.43 (-*C*-I), 24.79, 28.52, 28.68, 29.21, 29.55, 29.66, 29.72, 30.65, 33.72, 34.19 ppm. EI-MS: 356 (M^+^). Melting point: 38.7 ℃.

16-iodo-1-hexadecanethiol. ^1^H-NMR (400 MHz, CDCl_3_): δ 1.25-1.37 (m, 24H, -(*CH_2_)_12_*CH_2_CH_2_I), 1.60 (p, *J* =7.2 Hz, 2H, -*CH_2_*-CH_2_SH), 1.81 (p, *J* =6.9 Hz, 2H, -*CH_2_*-CH_2_I), 2.51 (q, *J* =7.2 Hz, 2H, -*CH_2_*-SH), 3.18 (t, *J* =6.9 Hz, 2H, -*CH_2_*-I) ppm. ^13^C-NMR (100 MHz, CDCl_3_): δ =7.36 (-*C*-I), 24.77, 28.51, 28.69, 29.20, 29.54, 29.64, 29.66, 29.71, 29.73, 29.76, 30.64, 33.71, 34.18 ppm. EI-MS: 384 (M^+^). Melting point: 47.3 ℃.

18-iodo-1-octadecanethiol. ^1^H-NMR (300 MHz, CDCl_3_): δ 1.25-1.37 (m, 28H, -(*CH_2_)_14_*CH_2_CH_2_I), 1.60 (p, *J* =7.2 Hz, 2H, -*CH_2_*-CH_2_SH), 1.82 (p, *J* =6.9 Hz, 2H, -*CH_2_*-CH_2_I), 2.52 (q, *J* =7.2 Hz, 2H, -*CH_2_*-SH), 3.18 (t, *J* =6.9 Hz, 2H, -*CH_2_*-I) ppm. ^13^C-NMR (75 MHz, CDCl_3_): δ =7.34 (-*C*-I), 24.79, 28.54, 28.70, 29.23, 29.57, 29.67, 29.69, 29.74, 29.76, 29.81, 30.67, 33.75, 34.20 ppm. EI-MS: 412 (M^+^). Melting point: 53.0 ℃.

**NMR spectra of HS(CH_2_)*_n_*X.** The NMR spectra for all the final thiol molecules are shown in Figs. S2-6.


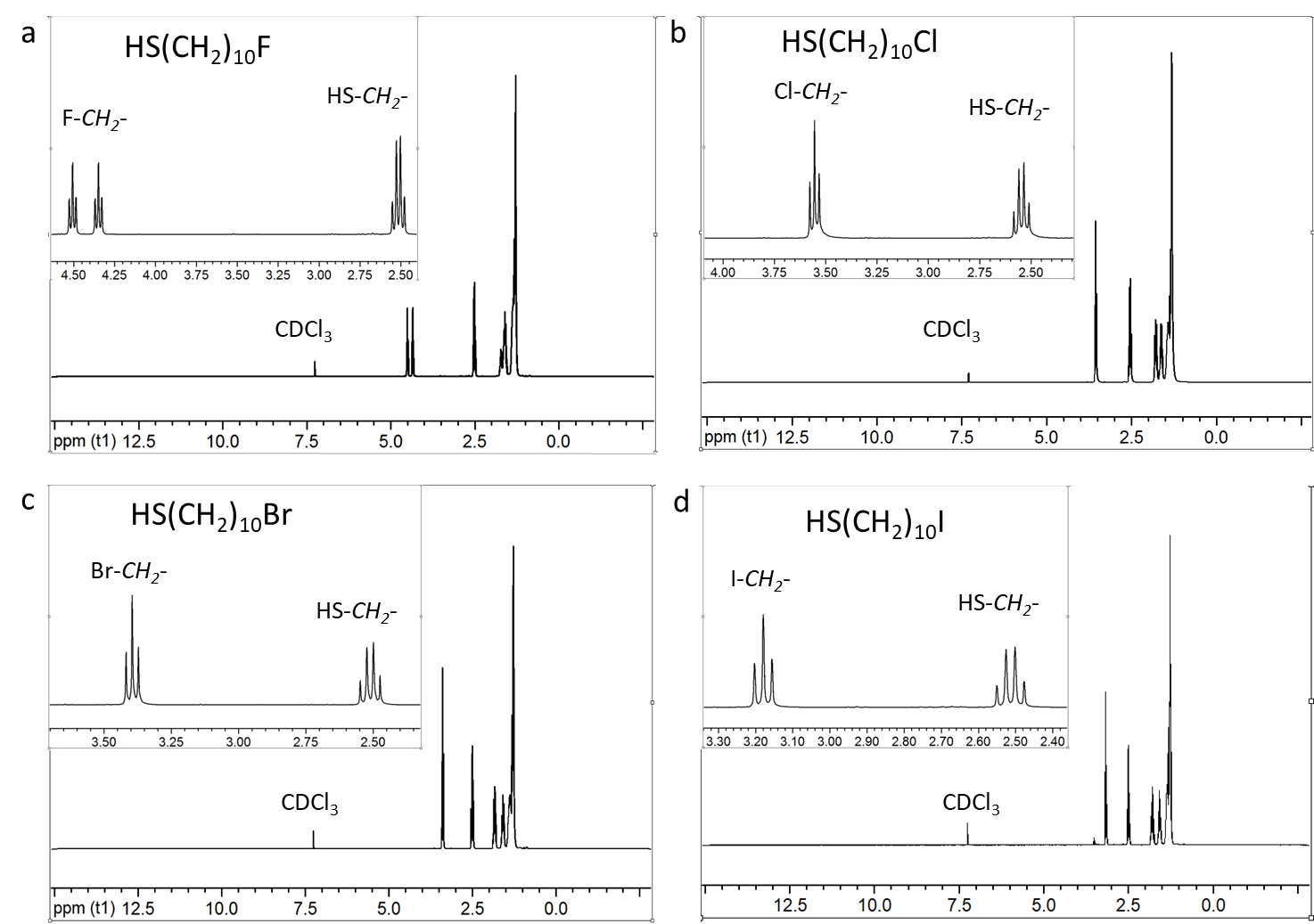


**Supplementary Fig. 2.** H^1^-NMR spectra of HS(CH_2_)_10_X, (**a**) for F, (**b**) for Cl, (**c**) for Br, (**d**) for I.


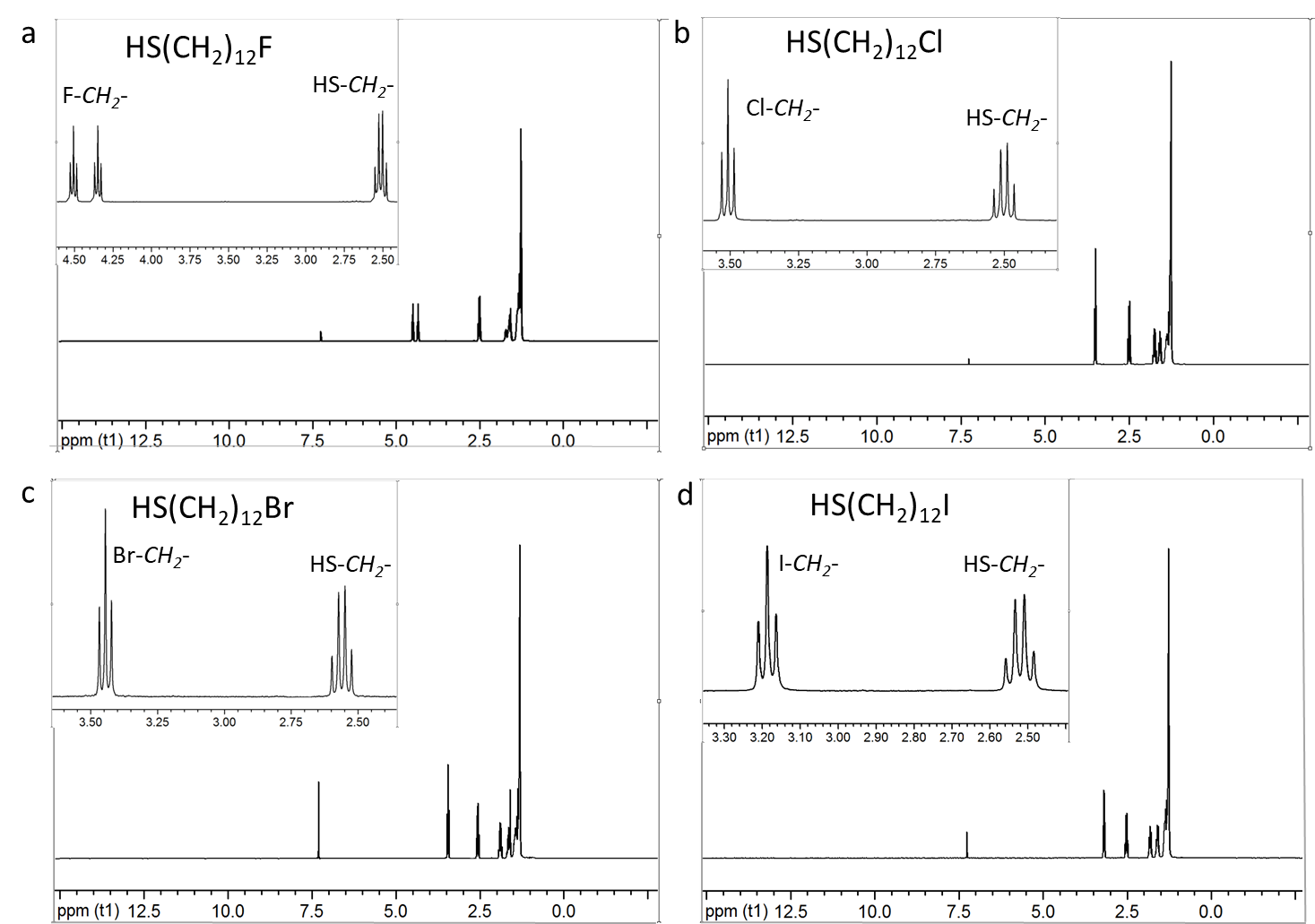


**Supplementary Fig. 3.** H^1^-NMR spectra of HS(CH_2_)_12_X, (**a**) for F, (**b**) for Cl, (**c**) for Br, (**d**) for I.

**
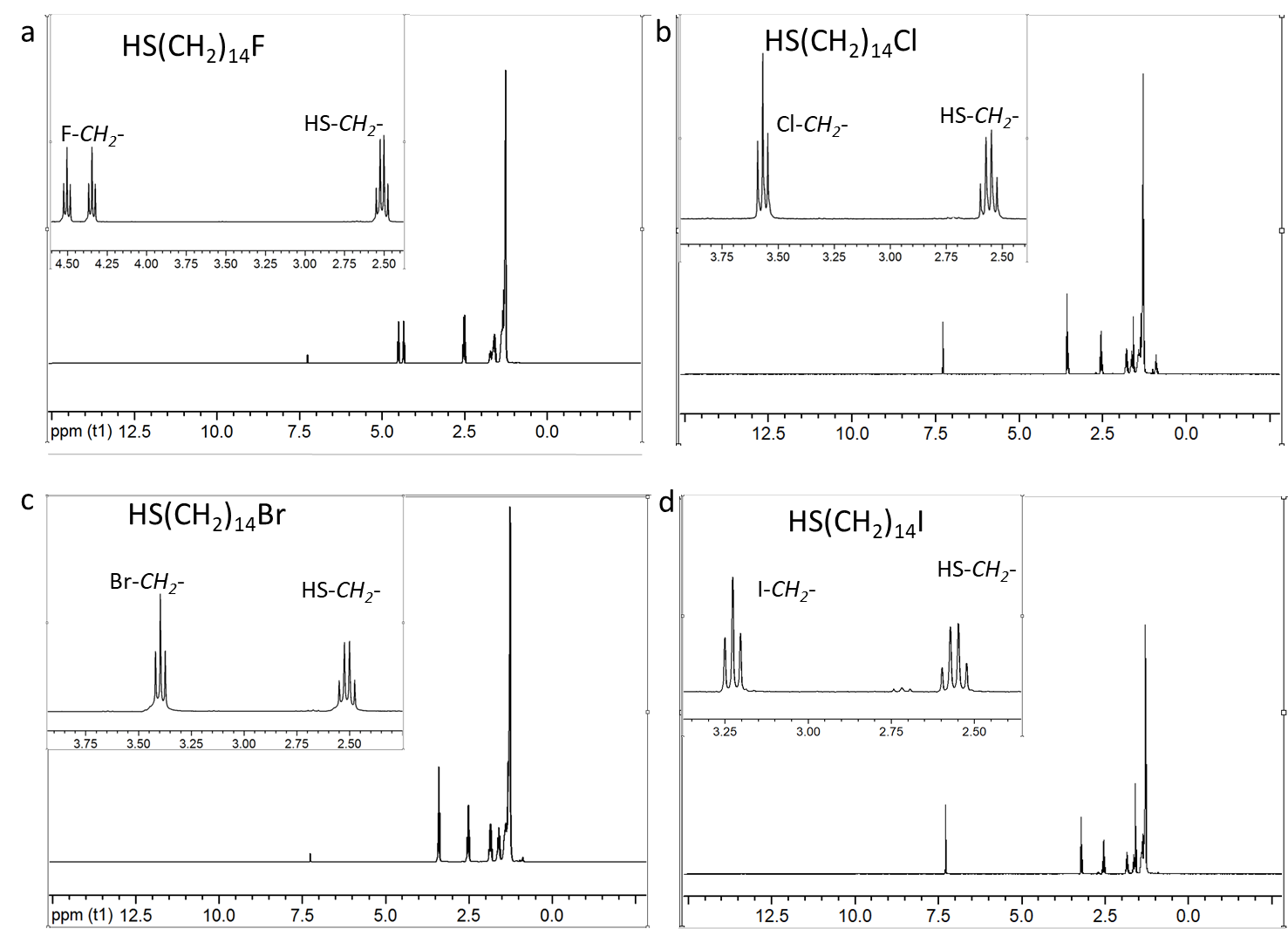
**

**Supplementary Fig. 4.** H^1^-NMR spectra of HS(CH_2_)_14_X, (**a**) for F, (**b**) for Cl, (**c**) for Br, (**d**) for I.


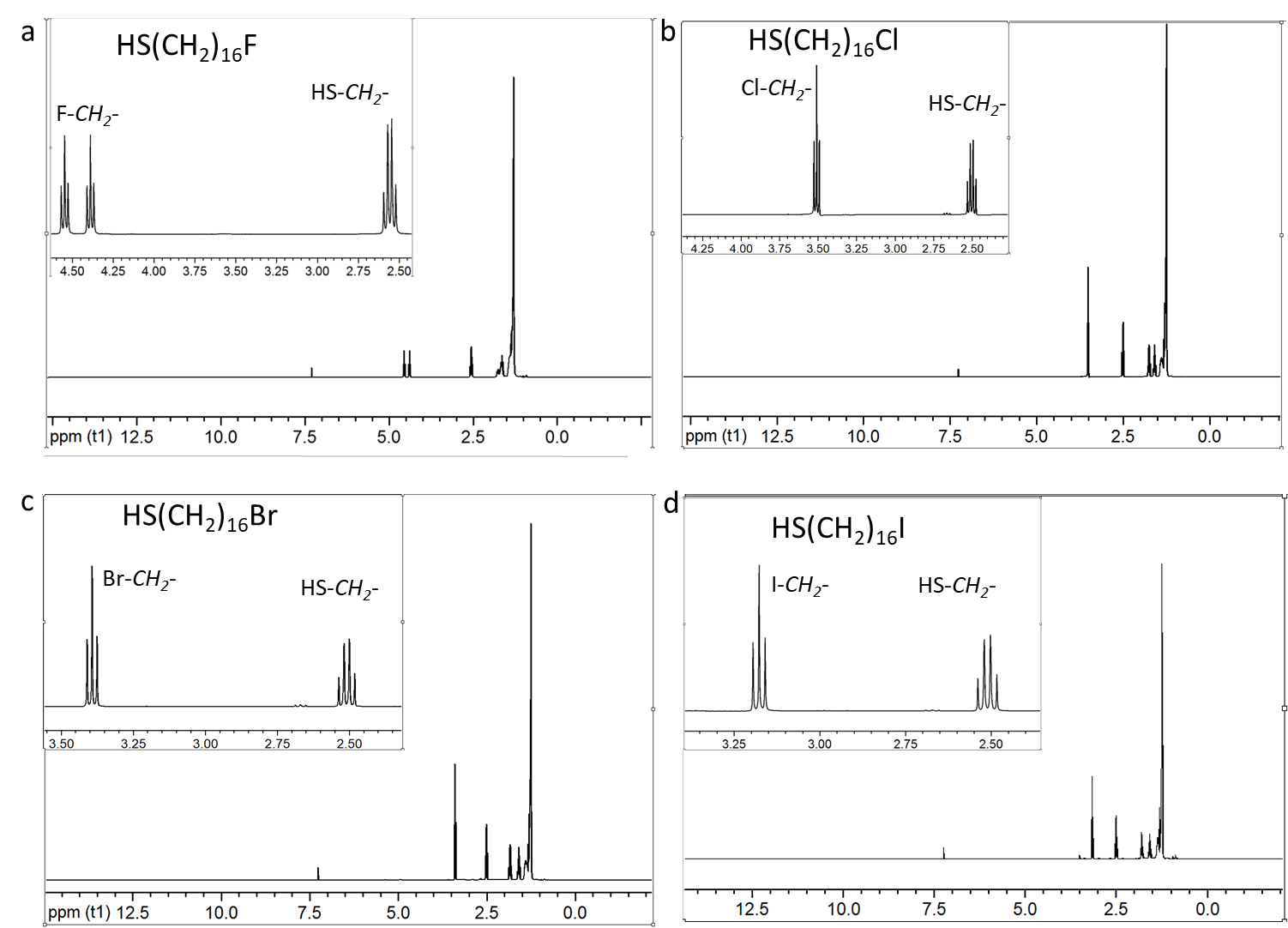


**Supplementary Fig. 5.** H^1^-NMR spectra of HS(CH_2_)_16_X, (**a**) for F, (**b**) for Cl, (**c**) for Br, (**d**) for I.


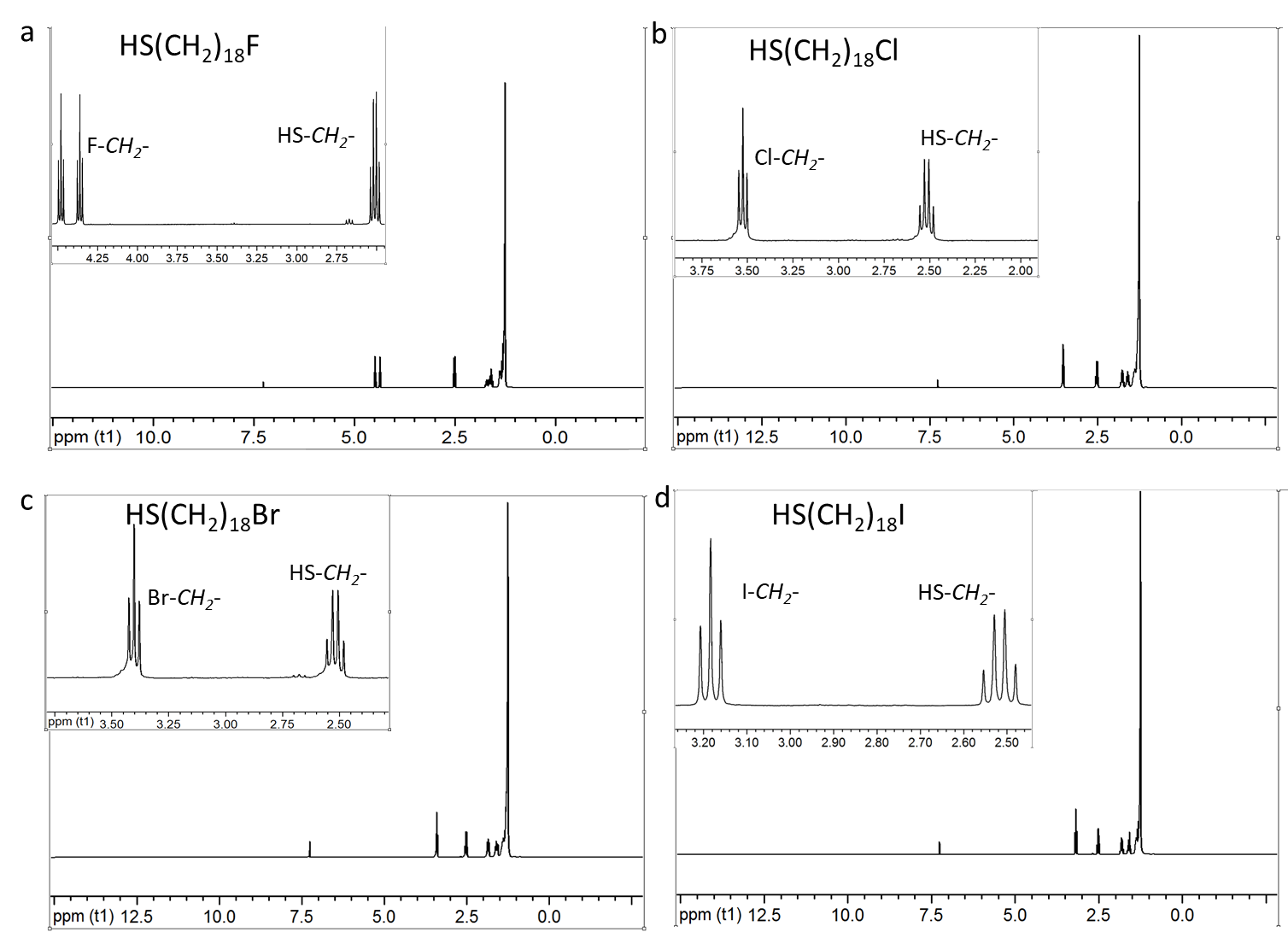


**Supplementary Fig. 6.** H^1^-NMR spectra of HS(CH_2_)_18_X, (**a**) for F, (**b**) for Cl, (**c**) for Br, (**d**) for I.

**Supplementary Section 3: SAM Preparation.**

The procedure of the preparation of the Ag surfaces and the SAMs were the same as before.^9^ Briefly, 200 nm Ag (with a purity of 99.99 %, purchased from ACI Alloy, USA) was deposited onto a clean 6 inch Prime Si (100) wafer (SYST Integration Pte Ltd, Singapore) under vacuum level of 5 × 10^-5^ Pa using a thermal evaporator (DZ270, SKY Technology Development Co., Ltd, Shenyang, China). The deposition rates were ~0.3 Å/s for the first 20 nm and ~1.0 Å/s for the remaining thickness. Piranha solution cleaned glass slides with dimensions of 1 × 2 cm were glued to the Ag surface using a thermal adhesive (EPOTEK 353ND, purchased from EPOXY TECHNOLOGY, INC. Massachusetts, USA), which was then cured at 80 ˚C using an oven (ZRD-A5110A, Zhicheng Inc. Shanghai, China) for 16 hours. The Ag substrates were stored in a clean dry box and the substrates were template-stripped just before use. The Ag substrates had an average rms roughness of 0.6 ± 0.1 nm over an area of 3 × 3 µm^2^. We prepared 3 mM SAM precursor-based ethanolic solutions using freshly distilled ethanol under N_2_. The Ag substrates were freshly template-stripped just before immersion into the ethanolic solution of the corresponding SAM precursor. The Ag substrates were left in the solutions for 3 h after which they were rinsed with approximately100 mL ethanol using a wash bottle and dried with N_2_ flow.

**Supplementary Section 4: SAM Surface Characterization.**

**ARXPS** **of the SAM**. We measured ARXPS at the Surface, Interface and Nanostructure Science (SINS) beam-line (a soft X-ray facility) located at the Singapore Synchrotron Light Source (SSLS).^10^ To avoid any contamination or unnecessary physisorption, the SAMs were freshly taken out of solution, just before loading the samples into the pre-chamber of the end-station of the SINS beamline. Throughout the measurements, the samples were kept under ultra-high vacuum (UHV) with pressure on the order of ~10^-9^ mbar. We report the ARXPS using two modes of the take-off angle (*θ*) (normal emission, *θ* = 90º and normal incidence, *θ* = 40º) that is defined as the angle between the axis of the analyser and the substrate surface. We report *Ψ*_SAM_ (nmol/cm^2^) and *d*_SAM_ (Ǻ) listed in Table 1 of the main text using a previously reported method.^1^ Briefly, *d*_SAM_ was calcuated using the effective intensities *I*_90º_ and *I*_40º_ of the S 2*p* spectra (at the peak centre of 162 eV), as shown in Eq. S1, *d*_1_ (=1.5 Å) is estimated from the radius of the S atoms and the S-C bond. To this *d*, Ag-S distance $d_{Ag-S}$ of 1.8 Å was added to get *d*_SAM_.

$d_{SAM} =\frac{\lambda\sin90^{\circ}\sin40^{\circ}\left[ \ln\left( \frac{I_{90^{\circ}}}{I_{40^{\circ}}} \right)+\ln\left( 1-e^{-\frac{d_{1}}{\lambda\sin40^{\circ}}} \right)-ln(1-e^{-\frac{d_{1}}{\lambda\sin90^{\circ}}}) \right]}{\sin90^{\circ}-\sin40^{\circ}}+d_{Ag-S}$ (S1)

We present the data in Figs. S7-9 as two sets of SAMs for the sake of comparison: C 1*s*, S 2*p*, Br 3*d*, Ag 3*d* for S(CH_2_)*_n_*Br (*n* = 10, 14, and 18) SAMs and C 1*s*, S 2*p*, Ag 3*d*, and the respective halogen spectra F 1*s*, Cl 2*p*, Br 3*d*, and I 3*d* of all S(CH_2_)_14_X SAMs. The peaks were fitted to Voigt functions (constant ratio of Lorentzian:Gaussian = 3:7) using XPSpeak 4.1 (Raymund Kwok, The Chinese University of Hong Kong, Shatin, Hong Kong) software and throughout the analysis, the spectra were corrected with a Shirley background. The errors from the peak fitting and experimental measurements are 10% in total.


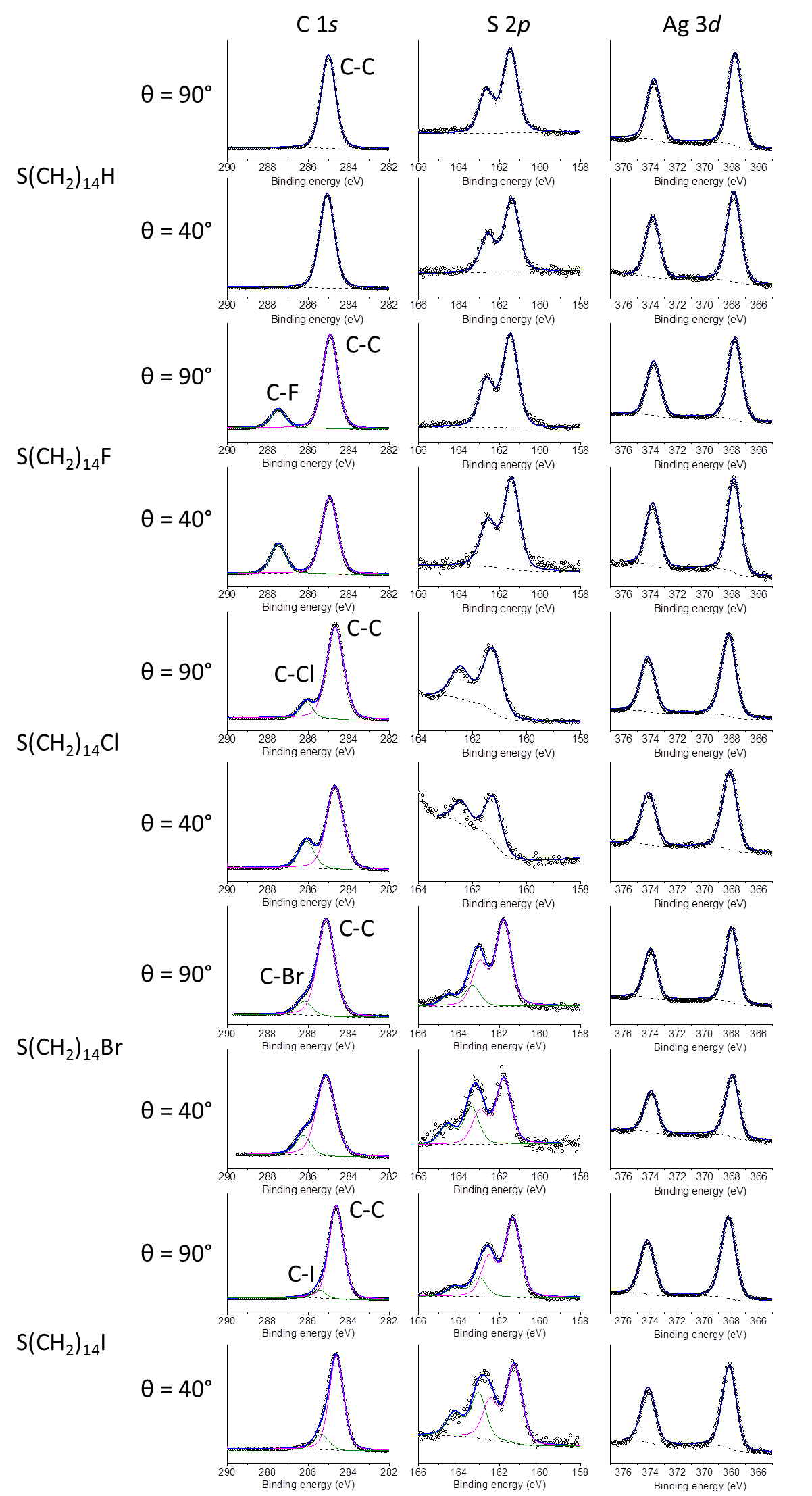


**Supplementary Fig. 7.**  Angle resolved C 1*s*, S 2*p*, and Ag 3*d* spectra recorded from S(CH_2_)_14_X SAMs.


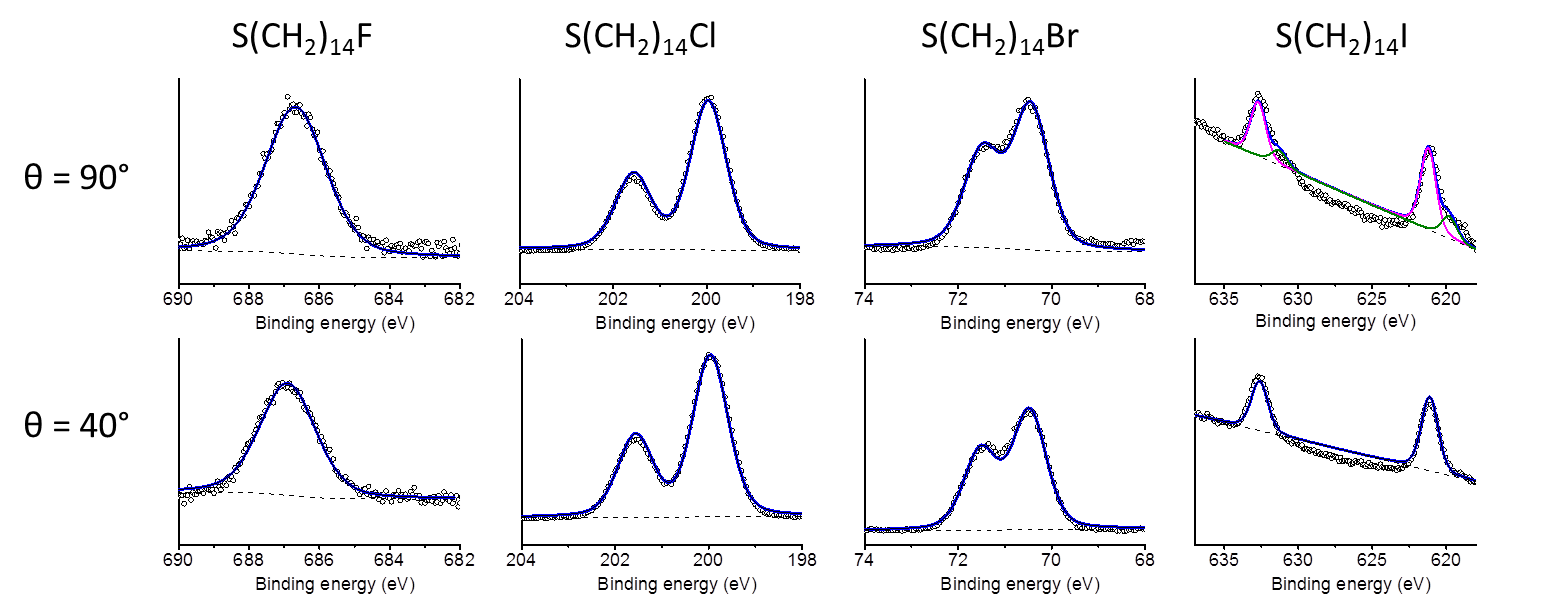


**Supplementary Fig. 8.**  Angle resolved F 1*s*, Cl 2*p*, Br 3*d*, and I 3*d* spectra recorded from S(CH_2_)_14_X SAMs.


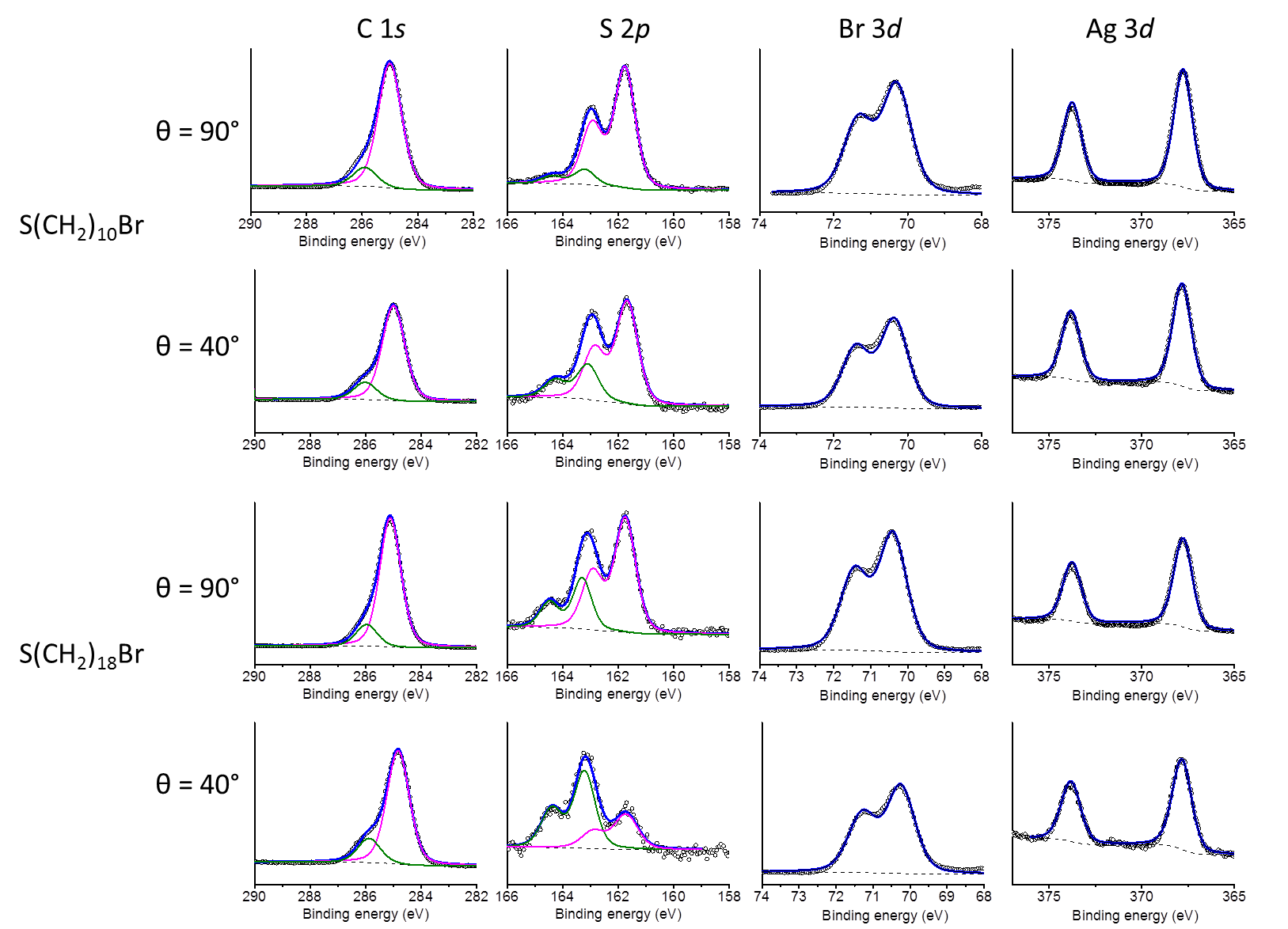


**Supplementary Fig. 9.**  Angle resolved C 1*s*, S 2*p*, Br 3*d*, and Ag 3*d* f spectra recorded from S(CH_2_)*_n_*Br SAMs (*n* = 10, 14, 18).

**UPS of the SAM**. We used a lab based UHV UPS system to determine work function and HOMO onset and from the secondary electron cut-off (SECO) and the beginning of the valence band respectively^11, 12^. The Fermi edges and work function values were calibrated against a reference, clean Ag surface (*Φ =* 4.26 eV). These spectra are shown in the Supplementary Fig. 10. The parameters are tabulated in Table 1 in the main text. All the UPS spectra were measured using He Iα light source and analysed using Omicron EA125 U7 hemispherical electron spectrometer with 7-channel detection installed on the VG Scientific ESCALab Mark 2 system.

**
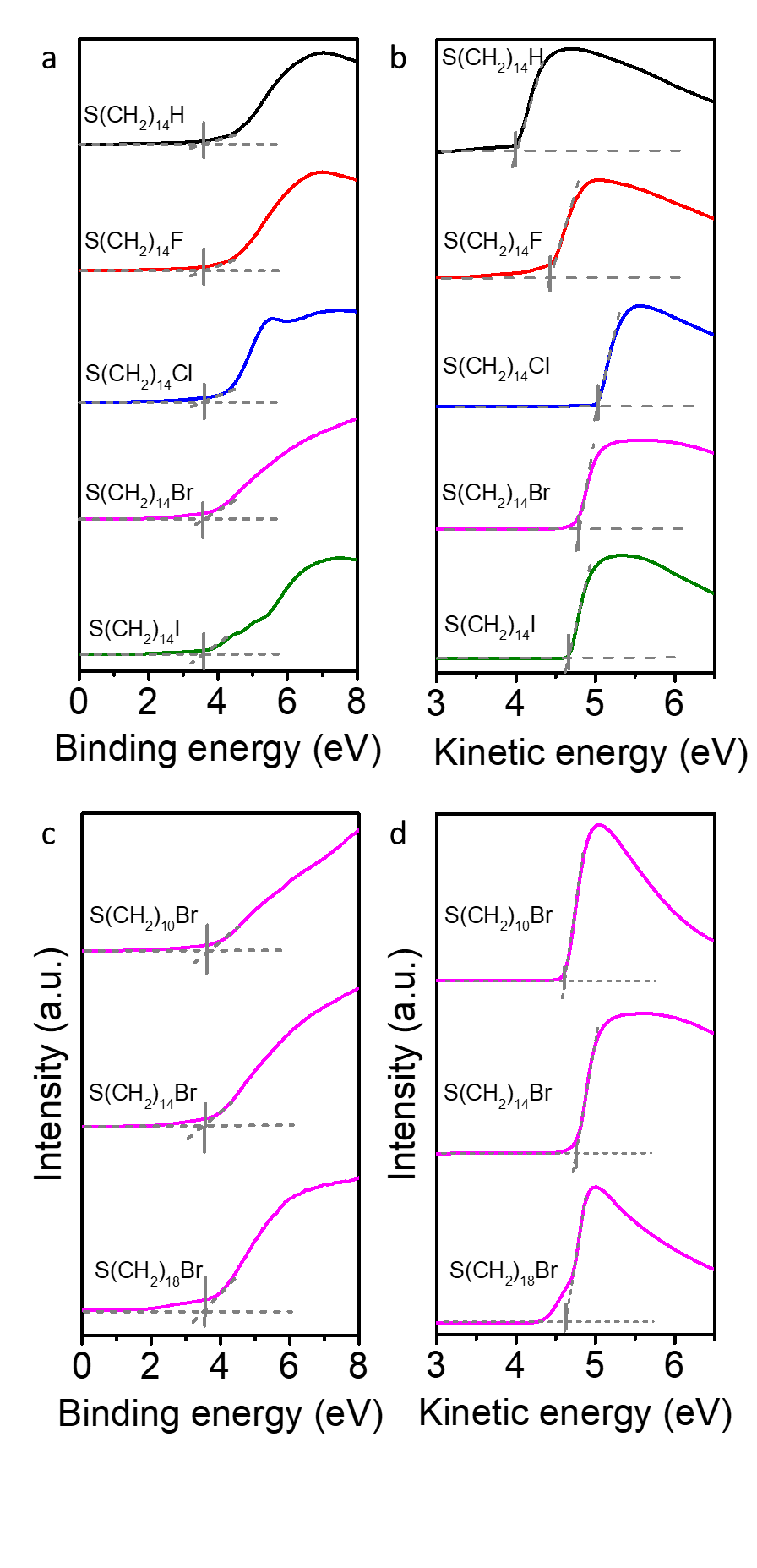
**

**Supplementary Fig. 10.**  Valence band spectra of S(CH_2_)*_n_*X SAMs on Ag. (**a** and **c**) The HOMO-onset was defined by the intercept of the grey dashed lines. (**b** and **d**) The SECO spectra, *Φ*_SAM_ was found at the intercept of the grey dashed lines.

**Supplementary Section 5: Molecular Dynamic (MD) Calculations.**

The MD *d*_SAM,MD_ for the Ag-S(CH_2_)*_n_*X SAMs  with *n* = 10, 12, 14, 16, or 18, and X = H, F, Cl, Br, or I are calculated. Values are time- and molecule-averages (in nm, with standard deviations underneath in parentheses) over 500 SAM structures each containing 128 molecules at a surface coverage of 1 nmol/cm^2^ on Ag(111), sampling every 100 ps during the final 50 ns of 100 ns of room temperature molecular dynamics.

**Supplementary Fig. 11.** The SAM thickness from molecular dynamics (MD). The error bars are shown in Supplementary Table 2. The slope of the linear fit to the data for X=Br (solid pink line) is 0.13 ± 0.01 nm per carbon (*R*^2^ = 0.99), which is close to that of 1.5 nm per carbon observed with ARXPS (Fig. 2**c**).

Supplementary Table 1. Summary of MD calculated *d*_SAM,MD_ (in Å) of S(CH_2_)*_n_*X molecules

| X | *n* = 10 | *n* = 12 | *n* = 14 | *n* = 16 | *n* = 18 |
| --- | --- | --- | --- | --- | --- |
| H | 14.9 ± 0.5 | 17.5 ± 0.6 | 20.4 ± 0.5 | 22.9 ± 0.5 | 25.6 ± 0.5 |
| F | 15.6 ± 0.4 | 18.1 ± 0.4 | 21.1 ± 0.3 | 23.7 ± 0.3 | 26.3 ± 0.3 |
| Cl | 16.0 ± 0.4 | 18.5 ± 0.4 | 21.5 ± 0.3 | 24.1 ± 0.3 | 26.6 ± 0.2 |
| Br | 16.2 ± 0.4 | 18.7 ± 0.4 | 21.7 ± 0.3 | 24.3 ± 0.3 | 26.8 ± 0.3 |
| I | 16.3 ± 0.4 | 18.9 ± 0.4 | 21.8 ± 0.3 | 24.4 ± 0.2 | 27.0 ± 0.2 |

**Supplementary Section 6: Electrical *J*(V) Characterization of the Molecular Junctions**

Here, we formed cone-shaped tips of GaO*_x_*/EGaIn which were then used to contact the SAMs followed by measuring the *J*(V) curves in the bias window of ±0.5 V following previous established procedures.^13^ In this paper, we used contact areas of ~350 μm^2^. Briefly, the bias was applied to the junction following the sequence of 0 → 0.5 → 0 → -0.5 → 0 V for each *J*(V) trace. For each type of junction, we measured a total of ~400 *J*(V) curves from ~20 junctions (with 20 traces per junction) fabricated on 3-4 samples. We determined the Gaussian log-average values of the current densities, <log_10_|*J*|>_G_, and the Gaussian log-standard deviation *σ*_log,G_ by plotting all measured values of *J* measured for each value of *V* in a histogram (Supplementary Figs. 12-16) to which we fitted a Gaussian function to obtain <log_10_|*J*|>_G_ and *σ*_log,G_. The results are summarized in Supplementary Tables 2-7. The junctions with the EGaIn stabilized in a micropore (see below) have a relatively large geometrical contact area of 960 μm^2^, but this method does not suffer from the significant leakage currents found in the cone-shaped tip junctions, which arise due to the need to push the EGaIn tip against the SAM until the desired geometrical contact area is obtained. For junctions with the EGaIn stabilized in a through-hole in PDMS, we only used junctions with *J*(V) characteristics within one log-standard deviation (σ_log,G_) of that obtained from the cone-shaped tip junctions in our *subsequent* measurements to ensure that the differences in junctions area did not cause any adverse effects due to leakage currents.^14, 15^


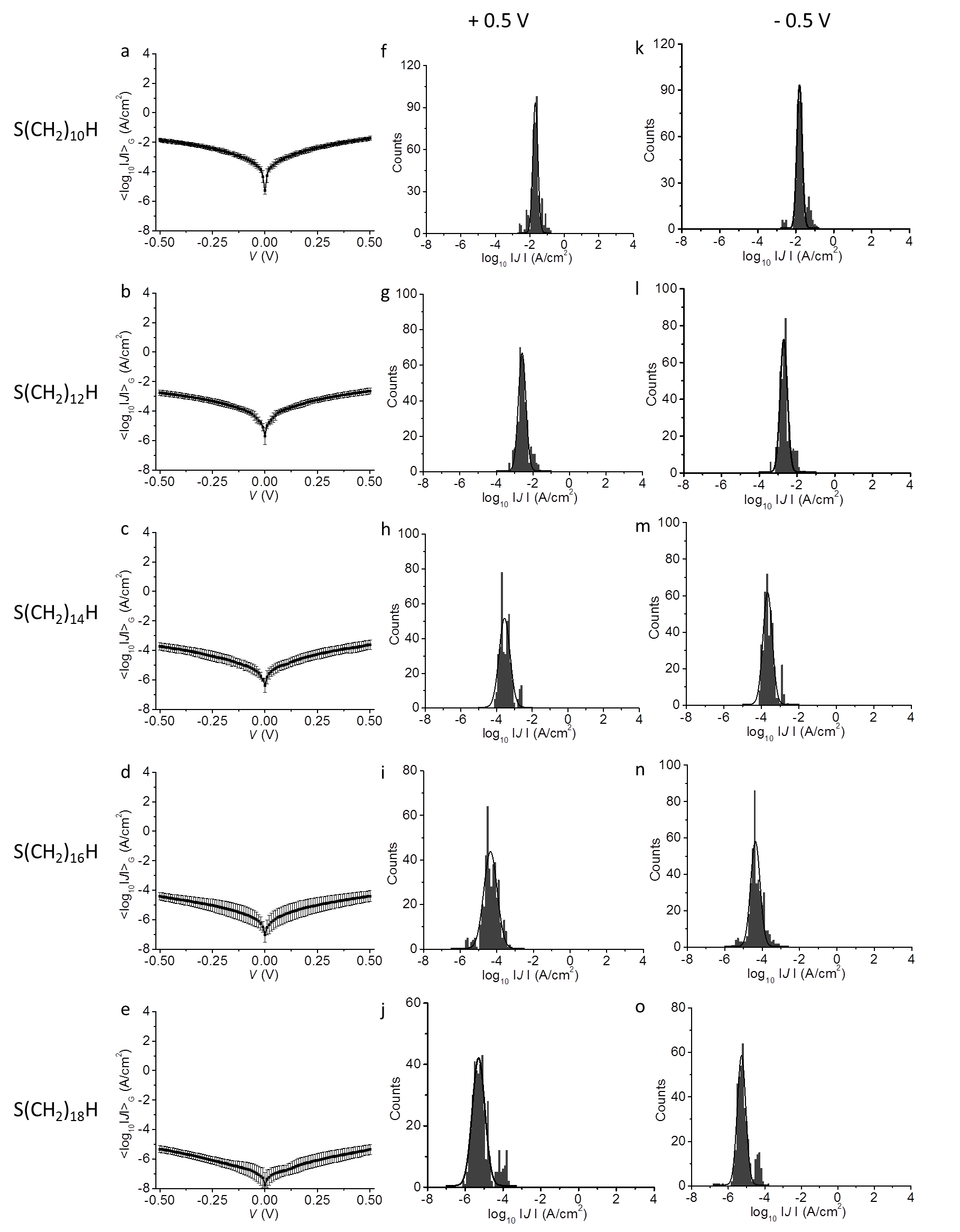


**Supplementary Fig. 12.** <log_10_|*J*|>_G_ *vs*. applied bias *V* curves (**a-e**) and histograms of log_10_|*J*| with a Gaussian fit (black lines) at +0.5 V (**f-j**) and –0.5 V (**k-o**) for Ag-S(CH_2_)*_n_*H//GaO*_x_*/EGaIn junctions. Error bars represent log-standard deviation from Gaussian fit (*σ*_log,G_) at each bias.

**
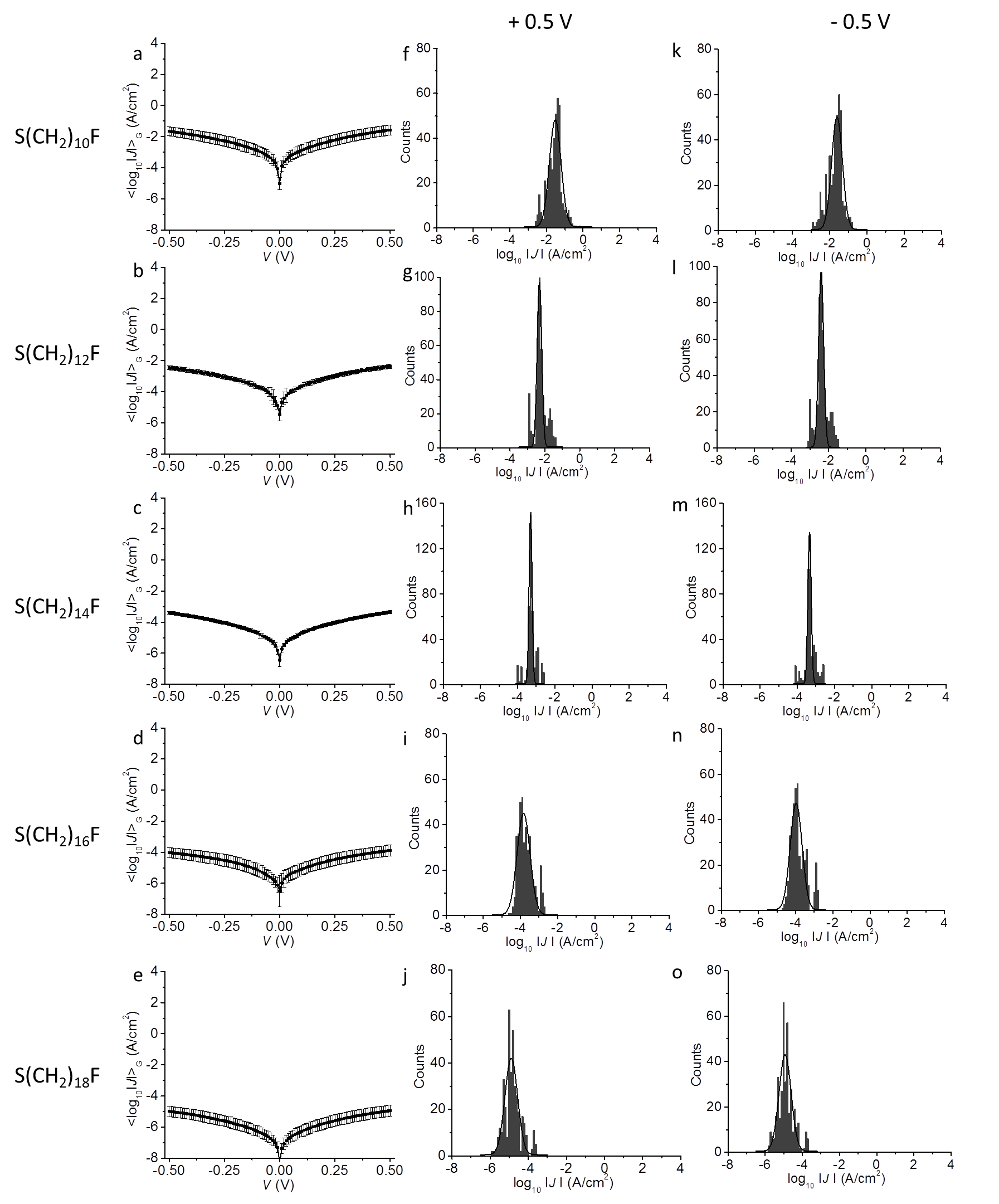
**

**Supplementary Fig. 13.** <log_10_|*J*|>_G_ *vs*. applied bias *V* curves (**a-e**) and histograms of log_10_|*J*| with a Gaussian fit (black lines) at +0.5 V (**f-j**) and –0.5 V (**k-o**) for Ag-S(CH_2_)*_n_*F//GaO*_x_*/EGaIn junctions. Error bars represent *σ*_log,G_ at each bias.


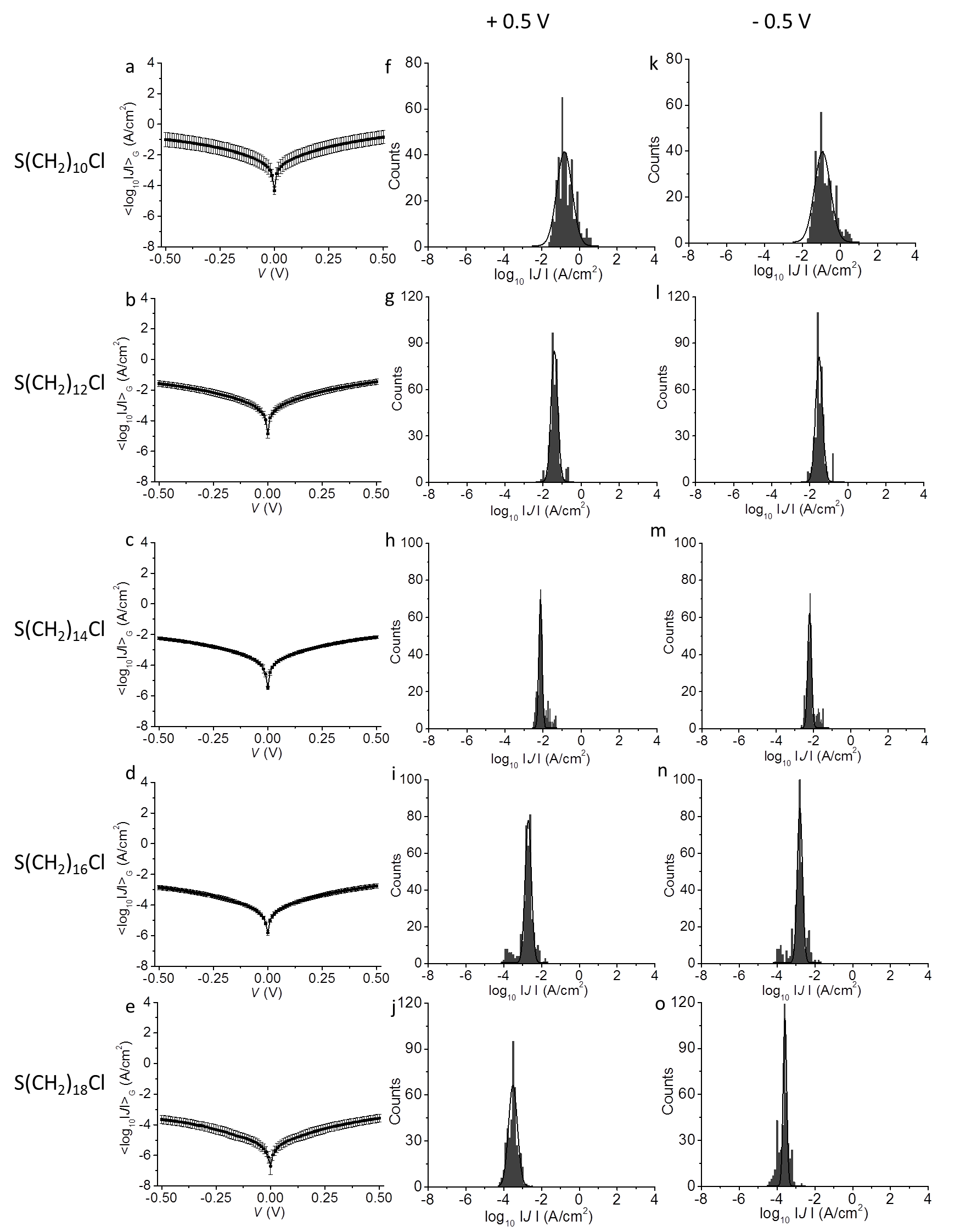


**Supplementary Fig. 14.** <log_10_|*J*|>_G_ *vs*. applied bias *V* curves (**a-e**) and histograms of log_10_|*J*| with a Gaussian fit (black lines) at +0.5 V (**f-j**) and –0.5 V (**k-o**) for Ag-S(CH_2_)*_n_*Cl//GaO*_x_*/EGaIn junctions. Error bars represent *σ*_log,G_ at each bias.


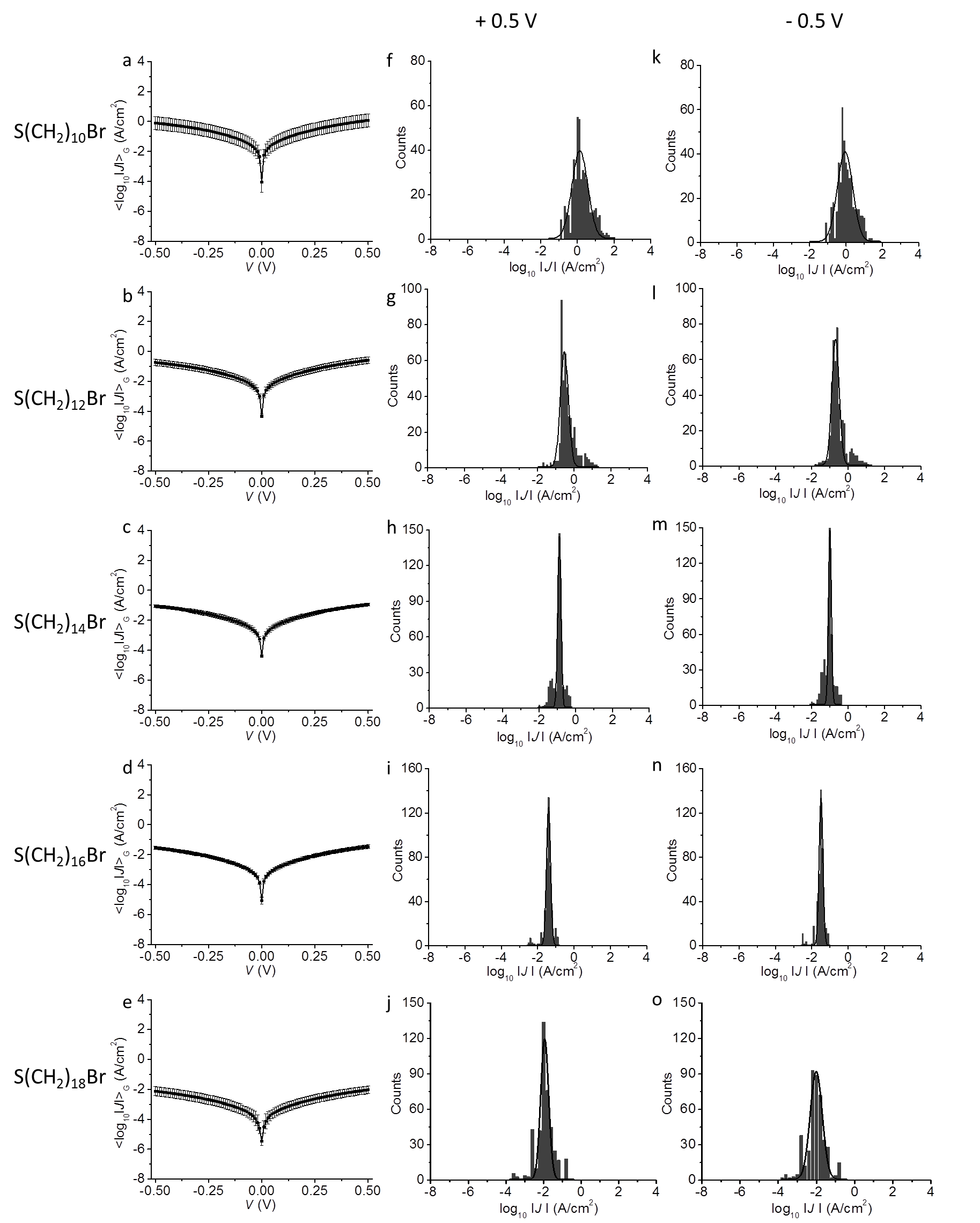


**Supplementary Fig. 15.** <log_10_|*J*|>_G_ *vs*. applied bias *V* curves (**a-e**) and histograms of log_10_|*J*| with a Gaussian fit (black lines) at +0.5 V (**f-j**) and –0.5 V (**k-o**) for Ag-S(CH_2_)*_n_*Br//GaO*_x_*/EGaIn junctions. Error bars represent *σ*_log,G_ at each bias.


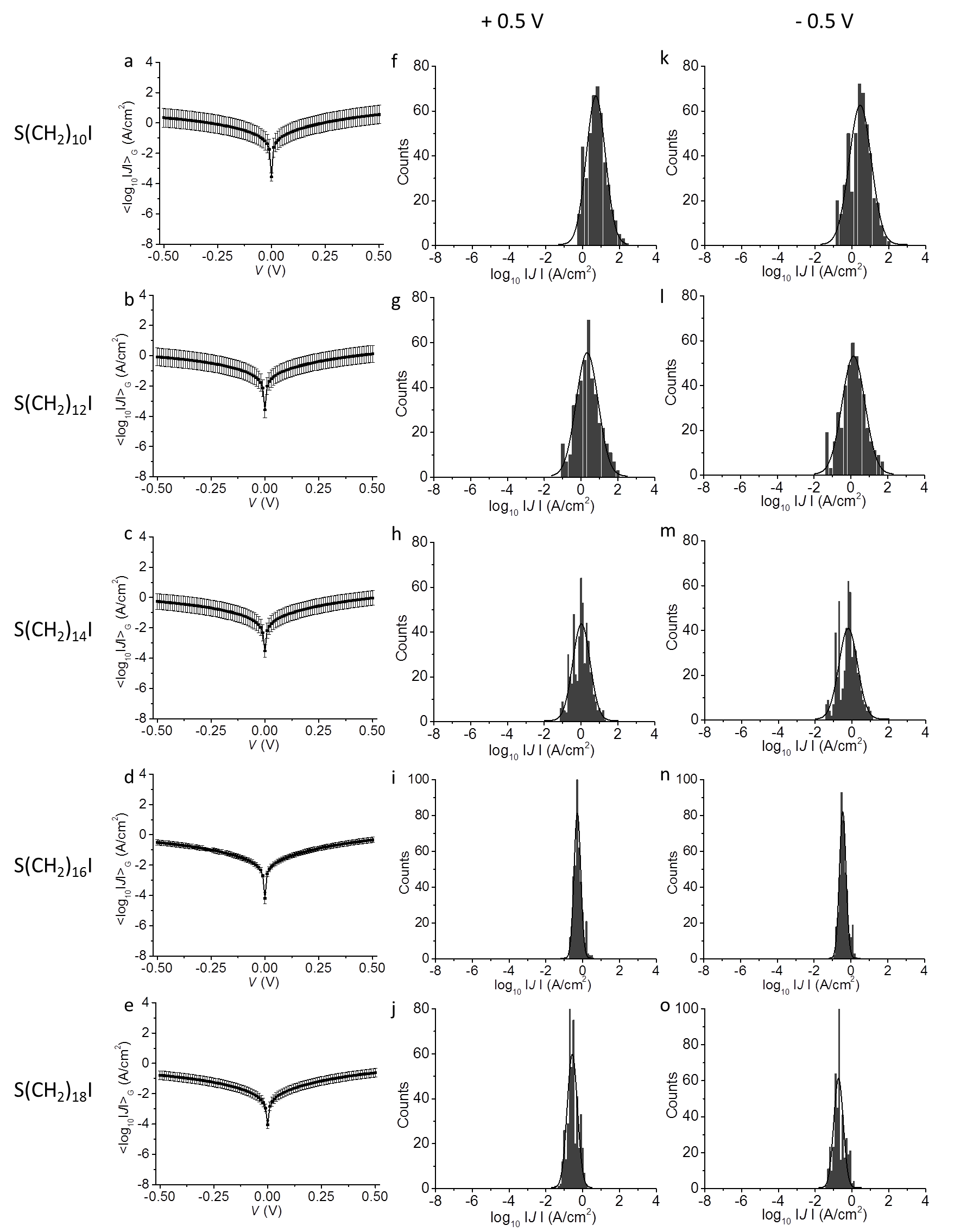


**Supplementary Fig. 16.** <log_10_|*J*|>_G_ *vs*. applied bias *V* curves (**a-e**) and histograms of log_10_|*J*| with a Gaussian fit (black lines) at +0.5 V (**f-j**) and –0.5 V (**k-o**) for Ag-S(CH_2_)*_n_*I//GaO*_x_*/EGaIn junctions. Error bars represent *σ*_log,G_ at each bias.


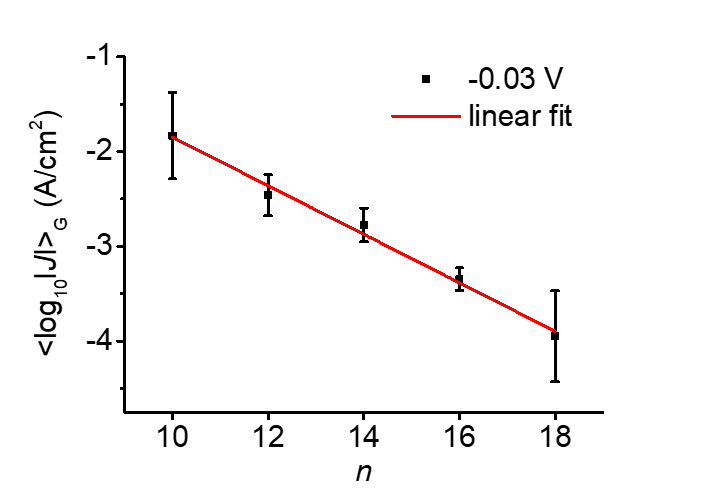


**Supplementary Fig. 17**. The plot of <log_10_|*J*|>_G_ *vs. n* for junctions of X=Br for *V* = -30 mV. The error bars represent the *σ*_log,G_, and the red line is fit to Eq. 1. The plot indicates *β* = 0.46 ± 0.03 Å^-1^ and log_10_|*J*_0_| = 0.71 ± 0.20 A/cm^2^ (*J*_0_ = 5.1 ± 2.0 A/cm^2^).

**Supplementary Fig. 18**. The plot of *β* vs X. The error bars of *β* represent the standard errors from fitting to data to Eq. 1. The dashed red line is a guide to the eye.

**Supplementary Table** **2**. Summary of the junctions of Ag-S(CH_2_)*_n_*H//GaO*_x_*/EGaIn junctions ±0.5 V

| S(CH_2_)*_n_*H | No. of  junctions | No. of  shorts/unstable junctions^a^ | No. of  traces^b^ | Yield  (%) | <log_10_\|*J*\|>_G_ (*σ*_log,G_) |
| --- | --- | --- | --- | --- | --- |
| 10 | 24 | 2 | 440 | 92 | -1.80 ± 0.14 |
| 12 | 19 | 1 | 380 | 95 | -2.70 ± 0.19 |
| 14 | 20 | 4 | 400 | 80 | -3.67 ± 0.25 |
| 16 | 21 | 1 | 420 | 95 | -4.37 ± 0.26 |
| 18 | 20 | 0 | 400 | 100 | -5.28 ± 0.23 |

^a^Short means the *J* is larger than the detection limit of our instrument (100 A/cm^2^); Unstable means the *J* increased or fluctuated suddenly by two orders of magnitude

^b^Number of traces from working junctions

**Supplementary Table** **3**. Summary of the junctions of Ag-S(CH_2_)*_n_*F//GaO*_x_*/EGaIn junctions ±0.5 V

| S(CH_2_)*_n_*F | No. of  junctions | No. of  shorts/unstable junctions | No. of  traces | Yield  (%) | <log_10_\|*J*\|>_G_ (*σ*_log,G_) |
| --- | --- | --- | --- | --- | --- |
| 10 | 25 | 3 | 440 | 88 | -1.61 ± 0.29 |
| 12 | 25 | 1 | 480 | 96 | -2.41 ± 0.13 |
| 14 | 27 | 4 | 460 | 85 | -3.34 ± 0.09 |
| 16 | 21 | 0 | 420 | 100 | -3.97 ± 0.32 |
| 18 | 19 | 0 | 380 | 100 | -4.93 ± 0.33 |

**Supplementary Table** **4**. Summary of the junctions of Ag-S(CH_2_)*_n_*Cl//GaO*_x_*/EGaIn junctions ±0.5 V

| S(CH_2_)*_n_*Cl | No. of  junctions | No. of  shorts/unstable junctions | No. of  Traces | Yield  (%) | <log_10_\|*J*\|>_G_ (*σ*_log,G_) |
| --- | --- | --- | --- | --- | --- |
| 10 | 24 | 3 | 420 | 88 | -0.93 ± 0.42 |
| 12 | 22 | 2 | 400 | 91 | -1.52 ± 0.18 |
| 14 | 23 | 2 | 420 | 91 | -2.21 ± 0.10 |
| 16 | 20 | 0 | 400 | 100 | -2.79 ± 0.14 |
| 18 | 26 | 4 | 440 | 85 | -3.59 ± 0.09 |

**Supplementary Table 5**. Summary of the junctions of Ag-S(CH_2_)*_n_*Br//GaO*_x_*/EGaIn junctions ±0.5 V

| S(CH_2_)*_n_*Br | No. of  junctions | No. of  shorts/unstable junctions | No. of  Traces | Yield  (%) | <log_10_\|*J*\|>_G_ (*σ*_log,G_) |
| --- | --- | --- | --- | --- | --- |
| 10 | 25 | 2 | 460 | 92 | -0.05 ± 0.42 |
| 12 | 24 | 2 | 440 | 92 | -0.69 ± 0.20 |
| 14 | 26 | 2 | 480 | 92 | -1.00 ± 0.08 |
| 16 | 23 | 2 | 420 | 91 | -1.49 ± 0.11 |
| 18 | 22 | 0 | 440 | 100 | -2.01 ± 0.31 |

**Supplementary Table 6**. Summary of the junctions of Ag-S(CH_2_)*_n_*I//GaO*_x_*/EGaIn junctions ±0.5 V

| S(CH_2_)*_n_*I | No. of  junctions | No. of  shorts/unstable junctions | No. of  Traces | Yield  (%) | <log_10_\|*J*\|>_G_ (*σ*_log,G_) |
| --- | --- | --- | --- | --- | --- |
| 10 | 28 | 5 | 460 | 82 | 0.45 ± 0.60 |
| 12 | 28 | 6 | 440 | 79 | 0.14 ± 0.62 |
| 14 | 28 | 2 | 520 | 93 | -0.21 ± 0.50 |
| 16 | 21 | 1 | 400 | 95 | -0.45 ± 0.18 |
| 18 | 25 | 3 | 440 | 88 | -0.74 ± 0.26 |

**Supplementary Table 7**. Summary of the *β* and log_10_|*J*_0_| of junctions of Ag-S(CH_2_)*_n_*X//GaO*_x_*/EGaIn junctions ±0.5 V.

| X | *β* (Å^-1^) | log_10_\|*J*_0_\| (A/cm^2^) |
| --- | --- | --- |
| H | 0.75 ± 0.04 | 2.5 ± 0.1 |
| F | 0.70 ± 0.02 | 2.6 ± 0.3 |
| Cl | 0.60 ± 0.03 | 2.6 ± 0.2 |
| Br | 0.39 ± 0.04 | 2.2 ± 0.2 |
| I | 0.25 ± 0.01 | 1.9 ± 0.1 |

**Temperature-dependent *J*(V) measurement**. The temperature-dependent *J*(V) measurement were conducted with top electrodes of polydimethylsiloxane (PDMS) filled with EGaIn in microfluidic channels.^16^ We placed the molecular junction in the chamber of a LakeShore probe station (Lake Shore Cryotronics, Inc.) which was connected to a chiller (Shelton Chillers, SAE-AC5, SHELTON(S) PTE LTD) and compressor (Sumitomo CRYOGENICS, F-50, Sumitomo Heavy Industries. Ltd.). The measurement was performed under a vacuum of 10^-6^ bar. The junction area of the devices with the EGaIn stabilized in microchannels in PDMS was 960 um^2^ and the junction area of the cone-shaped tip devices was 350-500 um^2^. We only used junctions with *J*(V) characteristics within one log-standard deviation (σ_log,G_) of that obtained from the cone-shaped tip junctions in our *J*(V,T) measurements to ensure that the differences in junctions area did not cause any adverse effects due to leakage currents.^14, 15^ The *J*(V) measurements were conducted over a temperature range of 340-250 K at intervals of 10 K. For each temperature, 10 traces were recorded, and the data were averaged and plotted in Fig. 3d.

**Supplementary Section 7: Electrical Impedance Characterization of the Molecular Junctions**

Impedance spectroscopy is important for understanding of the contribution of each electronic element in a molecular junction.^16, 17^ Under the disturbance of a sinusoidal voltage (*E*, in V) with a frequency *ω* (in rad/s, *ω* = 2π*f*, *f* is frequency, in Hz), the capacitor gives a capacitive reactance *X*_C_ of

*X*_C_ =1/*ω*C (S2)

Therefore, the impedance is more than the pure resistance of the circuit because it includes all the components that contribute to the complex impedance (*Z*, in Ω) under alternating voltage *E*:^18^

$E=E_{0}sin(\omega t)$ (S3)

The output of alternating current *I* (in A) is expressed with phase shift $ϛ$, in º, as:

$I=I_{0}sin(\omega t+ϛ)$ (S4)

The complex impedance *Z* is expressed with the real part of *Z* (*Z*´) and the imaginary part (*Z*"), and the modulus of the impedance (|*Z*|) is the output of the impedance device:

$Z=\frac{E}{I}=Z^{'}+jZ^{''}=|Z|e^{jϛ}$ (S5)

The molecular junctions can be modelled with the equivalent circuit as shown in Fig. 1a, for which $Z$ is given as^18^

$Z=\left( R_{C}+\frac{R_{SAM}}{1+\omega^{2}R_{SAM}^{2}C_{SAM}^{2}} \right)-j(\frac{\omega C_{SAM}R_{SAM}^{2}}{1+ \omega^{2}R_{SAM}^{2}C_{SAM}^{2}})$ (S6)

The values of *R*_C_, *R*_SAM_ and *C*_SAM_ were derived from Eq. S6, and their physical meanings are explained in the main text.

We used the Solartron 1296 DIELECTRIC INTERFACE and Solartron SI 1260 IMPEDANCE/GAIN-PHASE ANALYZER in our experiments. SMaRT v3.2.1 was used to control the impedance analyzer and collect data. ZView was used to analyze the impedance data. The AC bias applied was 30 mv for all junctions except for junctions with S(CH_2_)_14_F SAMs for which 50 mV amplitude was used. The sweep frequency was 100 to 1 × 10^6^ Hz with 10 frequencies/decade. The impedance was measured with 1.0 s integration and internal reference mode was chosen. For each kind of molecular junction, we measured 3 junctions (from each junction 3 spectra were recorded and averaged before fitting). We used equivalent circuit as shown in Fig. 1a to fit the impedance data after the data were checked with Kramers-Kronig transformations. Both the residual plots of the fits and Kramers-Kronig tests are shown below (Supplementary Figs. 19-22) and all *χ*^2^ values are given in Supplementary Table 8. The averaged values of the results and the standard deviations were obtained from 3 junctions for each kind of SAM. The Bode, Nyquist and phase vs. frequency plots are shown in Supplementary Figs. 23. Supplementary Tables 9-10 summarize the impedance results of the junctions.


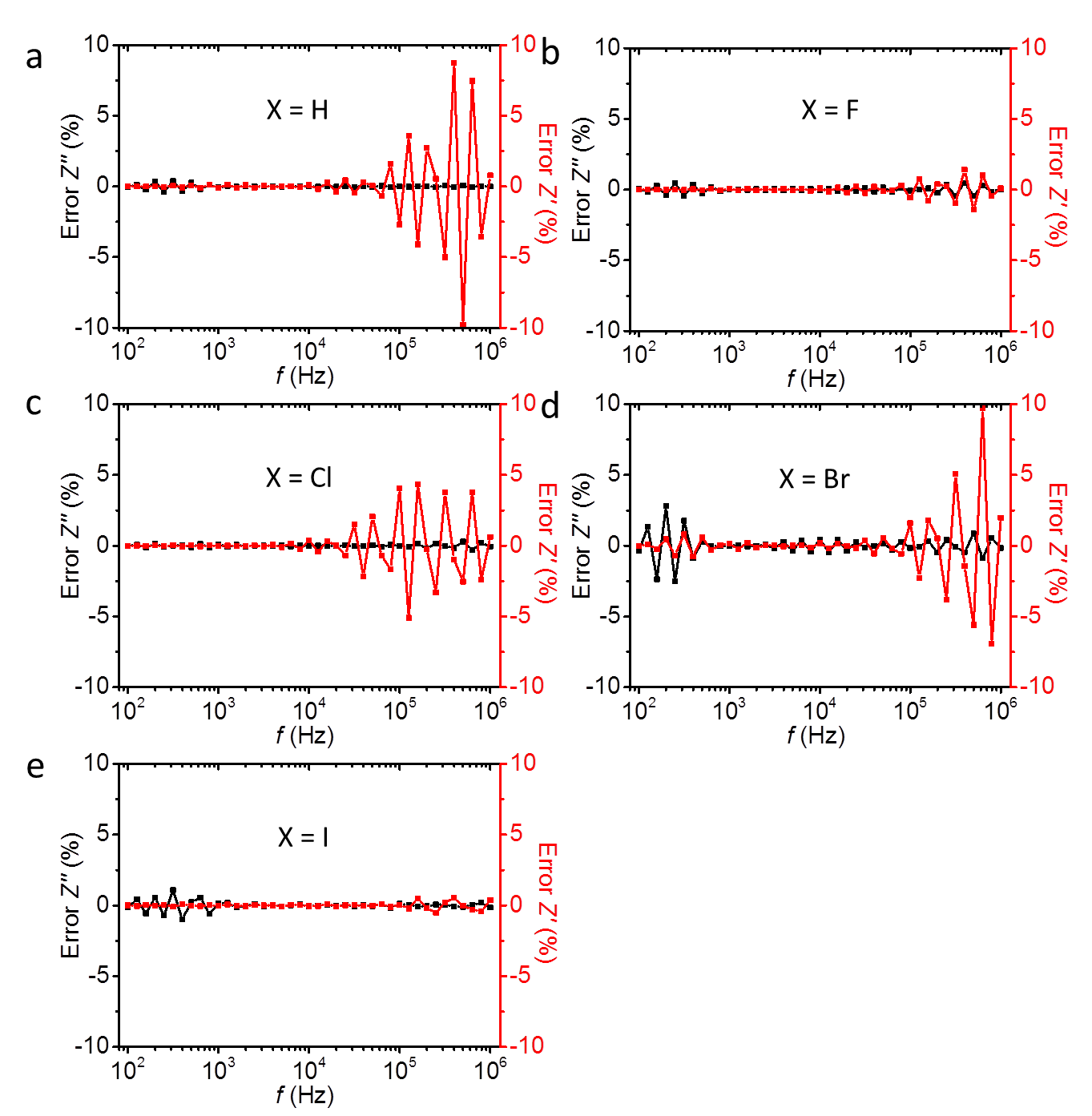


**Supplementary Fig. 19.**  The Kramers-Kronig residual plots of the experimental data for junctions of Ag-S(CH_2_)_14_X//GaO*_x_*/EGaIn. Panels **a-e** are for X= H to I, respectively.


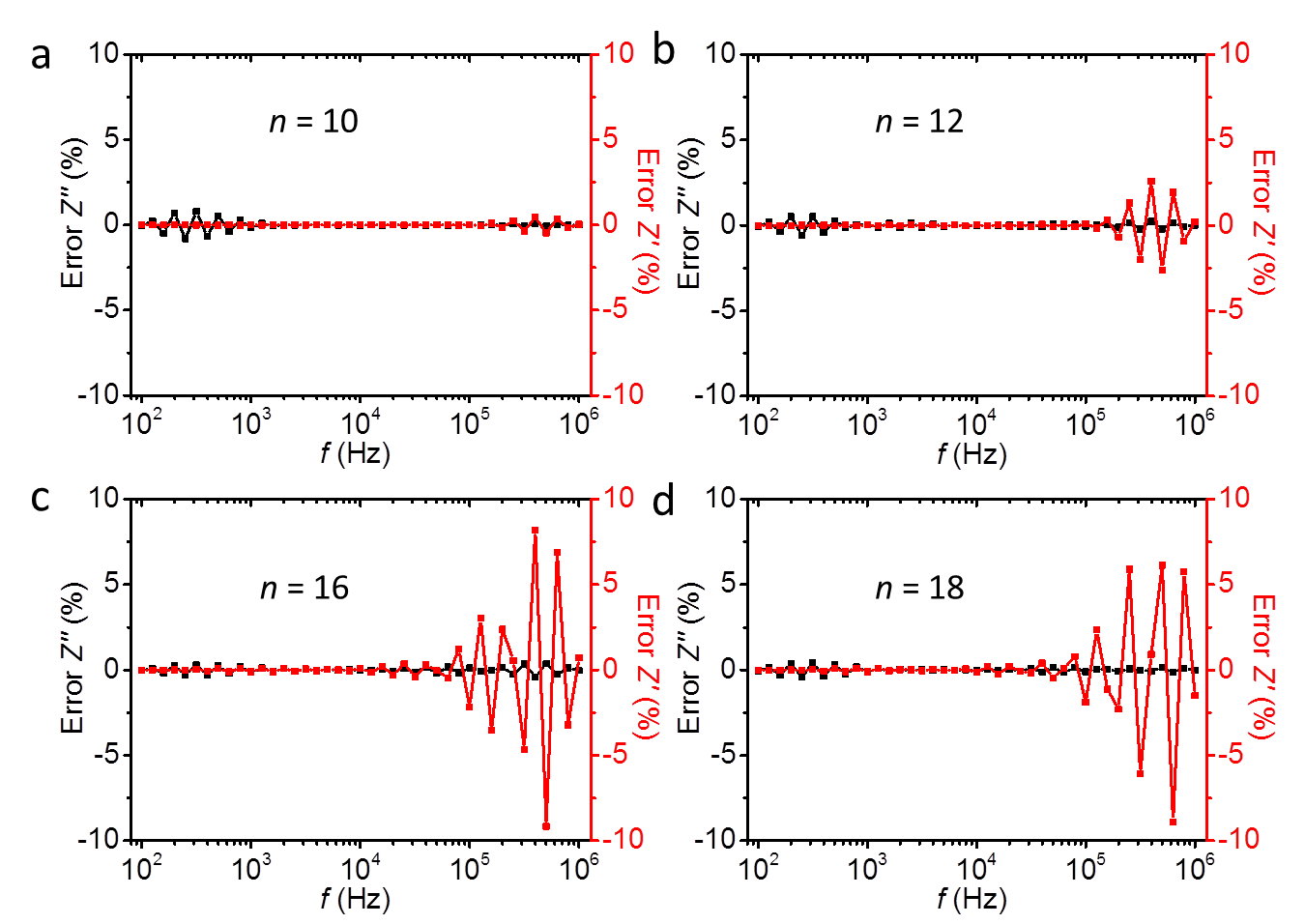


**Supplementary Fig. 20.**  The Kramers-Kronig residual plots of the experimental data for junctions of Ag-S(CH_2_)*_n_*Br//GaO*_x_*/EGaIn. Panels **a-d** are for *n* = 10, 12, 16 and 18, respectively. *n* = 14 is shown in Supplementary Fig. 19.

**
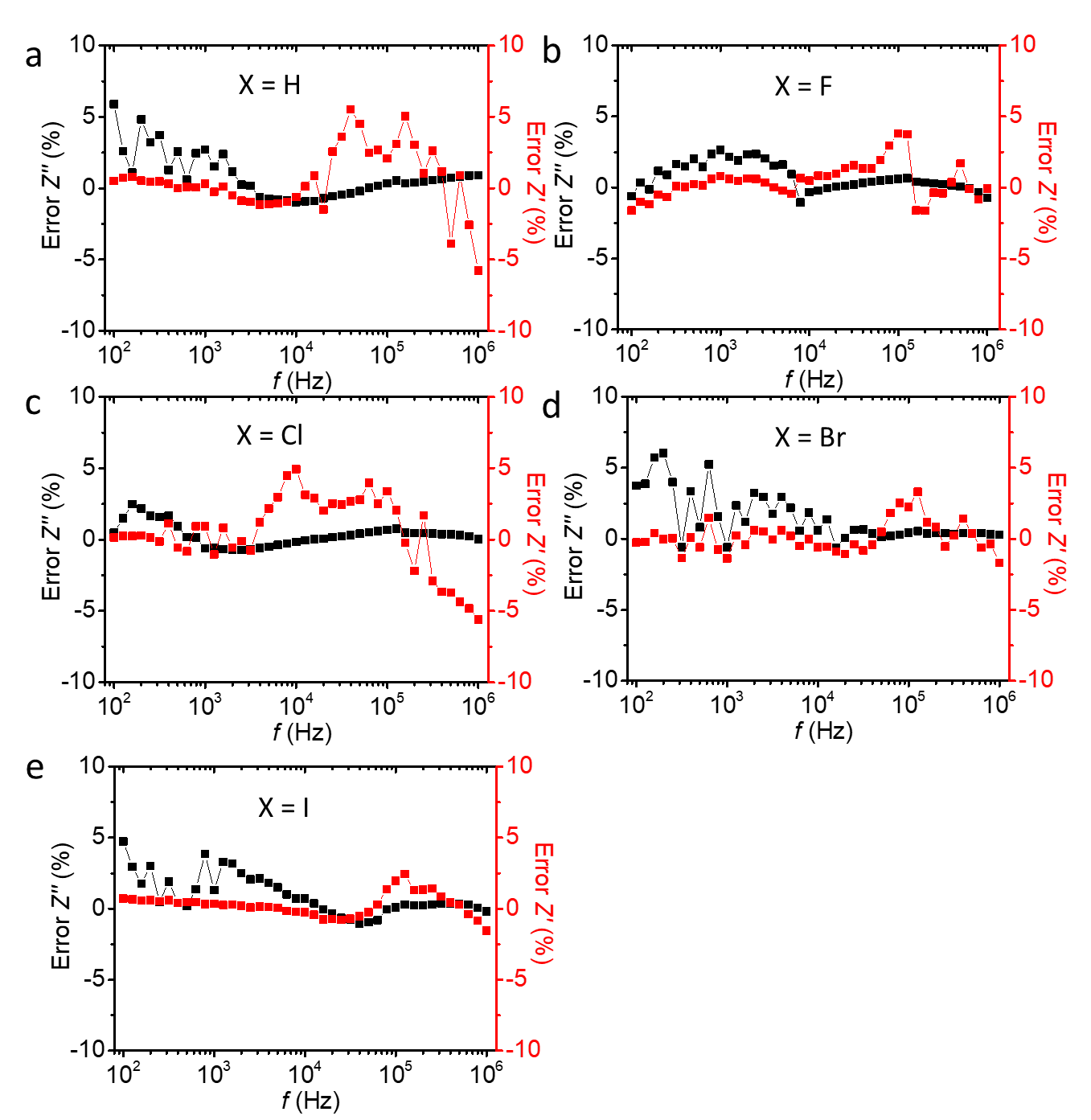
**

**Supplementary Fig. 21.**  Residual plots of the experimental data to the fit of the equivalent circuit shown in Fig. 1 for junctions of Ag-S(CH_2_)_14_X//GaO*_x_*/EGaIn. Panels **a-e** are for X= H to I, respectively.

**
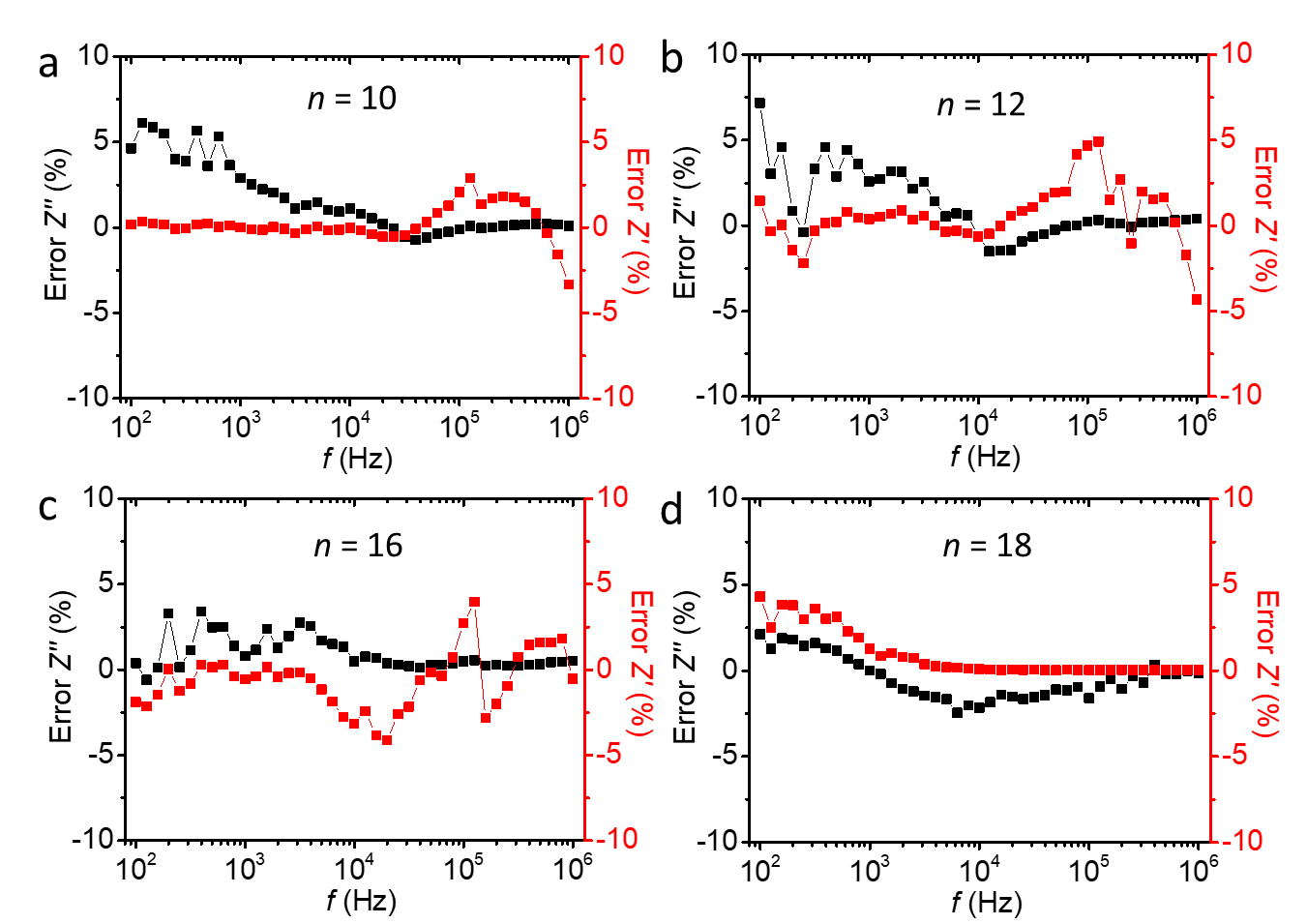
**

**Supplementary Fig. 22.**  Residual plots of the fits to the experimental data using the equivalent circuit shown in Fig. 1 for junctions of Ag-S(CH_2_)*_n_*Br//GaO*_x_*/EGaIn. Panels **a-d** are for *n* = 10, 12, 16 and 18, respectively. *n* = 14 is shown in Supplementary Fig. 21.

**Supplementary Table** **8**. χ^2^_KK_ and χ^2^_fit_ values

| X or *n* | χ^2^_KK_ | χ^2^_fit_ |
| --- | --- | --- |
| H | 0.0004 | 0.0005 |
| F | 0.0001 | 0.0002 |
| Cl | 0.0002 | 0.0006 |
| Br | 0.0003 | 0.0003 |
| I | 0.0001 | 0.0002 |
| 10 | 0.0001 | 0.0004 |
| 12 | 0.0001 | 0.0004 |
| 16 | 0.0003 | 0.0003 |
| 18 | 0.0003 | 0.0006 |

**
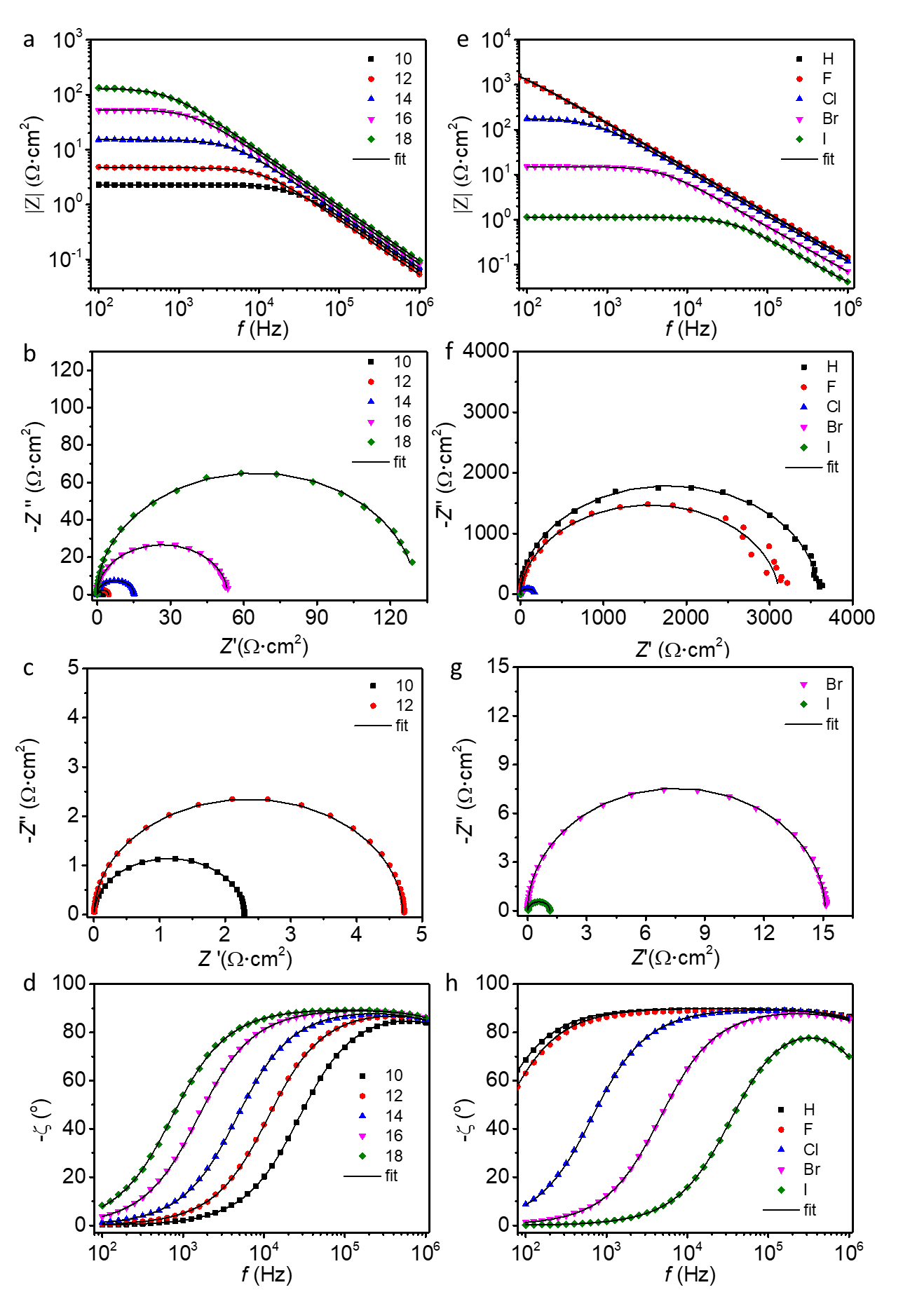
**

**Supplementary Fig. 23.** Bode plots (**a, e**), Nyquist plots (**b, c, f, g**), and the corresponding phase angle vs. frequency plots (**d, h**) of the Ag-S(CH_2_)*_n_*X//GaO*_x_*/EGaIn junctions. Panels **c** and **g** are the zoom-ins of **b** and **f**, respectively. The scatter symbols are the raw data, the black lines are fits to the equivalent circuit shown in Fig. 1.

**Supplementary Table** **9**. Summary of impedance results for junctions of Ag-S(CH_2_)_14_X//GaO*_x_*/EGaIn

| X | log_10_*R*_SAM_ (Ω•cm^2^) | *R*_C_ (mΩ•cm^2^) | *C*_SAM_ (µF/cm^2^) | *ε*_r_ |
| --- | --- | --- | --- | --- |
| H | 3.7 ± 0.1 | 19.6 ± 1.3 | 1.3 ± 0.2 | 2.9 ± 0.3 |
| F | 3.4 ± 0.1 | 20.5 ± 1.1 | 1.1 ± 0.3 | 2.5 ± 0.6 |
| Cl | 2.3 ± 0.4 | 9.2 ± 0.1 | 1.3 ± 0.1 | 3.0 ± 0.2 |
| Br | 1.0 ± 0.1 | 6.2 ± 1.1 | 2.1 ± 0.4 | 4.7 ± 0.9 |
| I | 0.21 ±0.08 | 3.5 ± 0.3 | 4.1 ± 0.7 | 8.9 ± 1.6 |

**Supplementary Table** **10**. Summary of impedance results for junctions of Ag-S(CH_2_)*_n_*Br//GaO*_x_*/EGaIn

| *n* | log_10_*R*_SAM_ (Ω•cm^2^) | *R*_C_ (mΩ•cm^2^) | *C*_SAM_ (µF/cm^2^) | *ε*_r_ |
| --- | --- | --- | --- | --- |
| 10 | 0.36 ± 0.11 | 5.4 ± 0.5 | 2.8 ± 0.2 | 4.4 ± 0.4 |
| 12 | 0.67 ± 0.10 | 5.5 ± 2.0 | 2.4 ± 0.2 | 4.5 ± 0.4 |
| 14 | 1.0 ± 0.10 | 6.2 ± 1.1 | 2.1 ± 0.4 | 4.7 ± 0.9 |
| 16 | 1.5 ± 0.09 | 6.2 ± 0.5 | 1.8 ± 0.1 | 4.4± 0.3 |
| 18 | 2.1 ± 0.09 | 6.5 ± 0.7 | 1.7 ± 0.1 | 4.6 ± 0.2 |

**Supplementary Section 8: DFT Calculations**

We performed density functional theory (DFT) calculations as implemented in the VASP code^19^ using a plane-wave basis set (cut-off energy: 300 eV) and the projector augmented wave (PAW) method^20^. We employed the Perdew-Burke-Enzerhof (PBE) exchange-correlation functional within the generalized gradient approximation^21^. To sample the Brillouin zone, we used a *Γ*-centered k-point grid of 8×8×1 points. We used a 3×2√3 Ag surface unit cell with a PBE optimized lattice constant of 4.14 Å and four molecules arranged in a herringbone pattern per unit cell (see Supplementary Fig. 24). Similar structures have been reported for alkyl-thiol SAMs on Ag and Au.^22^ Note that we performed calculations on different herringbone structures (e.g., different relative orientation of the molecules), all of which are very close in energy after structural optimizations. Hence, we chose the structure for which the work-functions were closest to the experimental values. The structures of the SAMs were optimized using GADGET^23^.


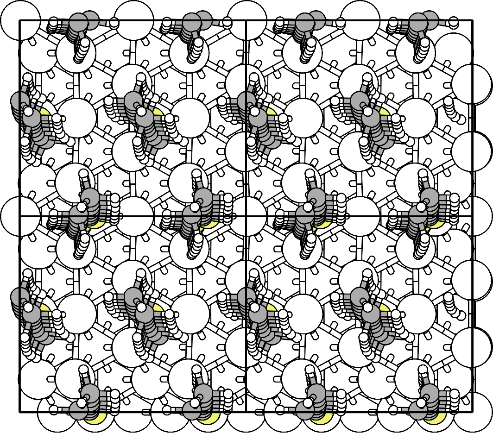


**Supplementary Fig. 24.** Top view of herringbone structure for Ag-S(CH_2_)_14_-H. The black solid lines indicate the unit cell (four cells are shown here to visualize the herringbone structure).

The dielectric constants were calculated for the free-standing monolayers using the procedure by Heitzer et al.^24^ except for the definition of the thickness of the monolayer, which we calculated based on vdW radii as suggested by Romaner et al.^25^ The vdW radii for the different elements were taken from Ref 26. For these calculations we used a H termination for the thiol group. The S-H bond was optimized using GADGET, keeping all atoms but the H fixed. For the calculation of the dielectric constants we applied an electric field of ±0.001 a.u. Supplementary Table 11 shows the dielectric constants calculated with and without inclusion of the vdW radii of the atoms in the thickness definition. We note that unfortunately in Ref. 1 the DFT calculations were incorrect and happened to agree with experiment due to error cancellation, which was not realized at the time. The authors responsible for the DFT calculations in the present work (who were not involved in the previous study) found that the mistake was based on not including a dipole correction in this earlier work, which would have been of utmost importance to obtain reliable dielectric constants. In short, accurate electronic structure calculations of a polar 2D slab or thin film in a 3D simulation cell must correct for differences in the vacuum potentials on both sides of the slab or film to avoid spurious electrostatic effects. This is known as a “dipole correction” and must be included in periodic DFT calculations of such systems. However, in Ref. 1 this was not done and, therefore, the field-induced dipole moments were significantly overestimated. As a consequence, the dielectric constants reported previously were incorrect and disagree with the values reported in our current study.

**Supplementary Table 11**: Dielectric constants from DFT with and without inclusion of the vdW radii in the thickness definition.

| X | ε_vdW_ | ε_atomic_ |
| --- | --- | --- |
| H | 2.2 | 2.5 |
| F | 2.1 | 2.5 |
| Cl | 2.2 | 2.7 |
| Br | 2.3 | 2.8 |
| I | 2.4 | 3.0 |

Supplementary Figs. 25 and 26 show the charge densities of the HOMOs and LUMOs of the SAMs with the different terminations as calculated from DFT with VASP.

**
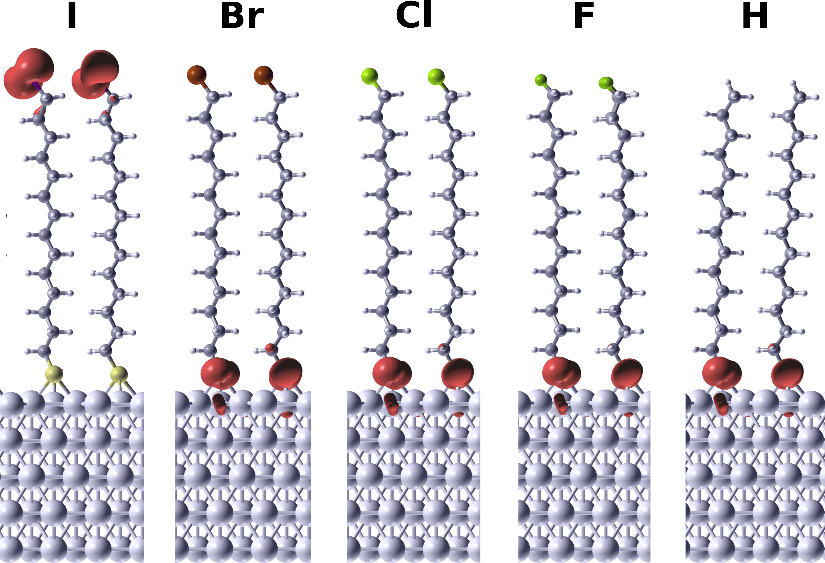
**

**Supplementary Fig. 25**. Charge densities of the highest occupied state for the Ag-(CH_2_)_14_-X SAMs, with X=I, Br, Cl, F, H, plotted as iso-surfaces (red). This state is mostly localized on the S atom, except for X=I and Br where it overlaps with the molecular HOMO. The iso-level was set to 0.02 e/Å^3^.

**
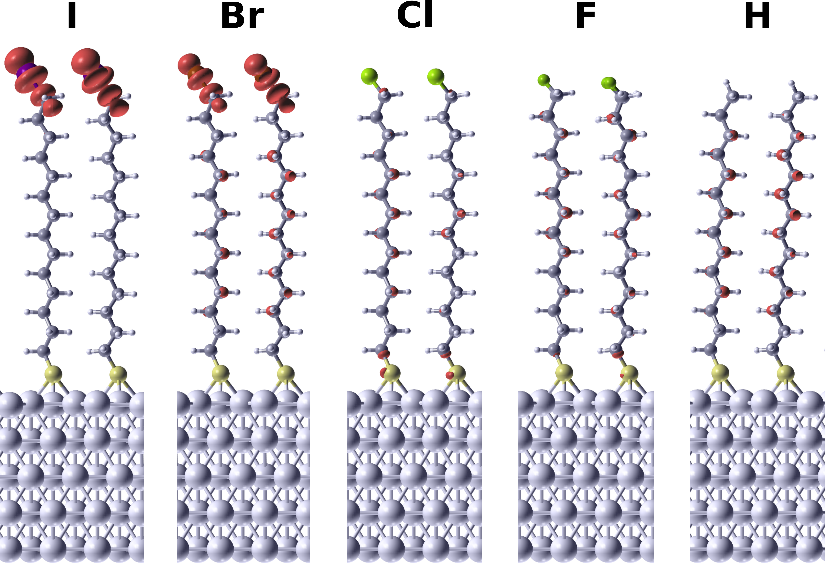
**

**Supplementary Fig. 26**. Charge densities of the LUMO for the Ag-(CH_2_)_14_-X SAMs, with X=I, Br, Cl, F, H, plotted as iso-surfaces (red). This state is mostly localized on the X for X=I, Br, Cl, but gets more delocalized over the whole backbone for X=F and H. The iso-level was set to 0.01 e/Å^3^.

**Calculations for *n*=10, with X=H and X=I**


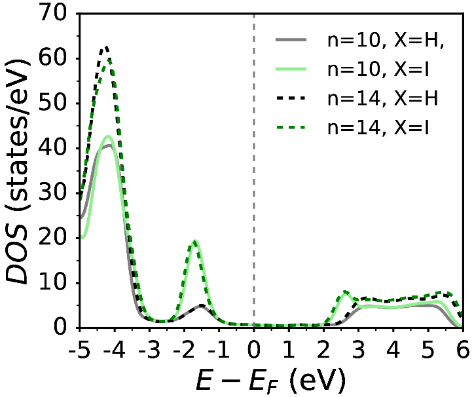
The differences in the electronic structure between Ag-(CH_2_)_10_-X and Ag-(CH_2_)_14_-X are minor. While the dielectric constants are 0.1 lower for *n*=10 than for *n*=14, the work-functions are slightly (~0.05 eV) larger for the shorter chains. The differences in the DOS are of the same order of magnitude as in the work-functions (see Supplementary Fig. 27).

**Supplementary Fig. 27**. DOS projected onto the organic part of Ag-(CH_2_)_10_-X and Ag-(CH_2_)_14_-X SAMs.

**Coordinates of relaxed structure with X=H in the VASP input format (POSCAR):**

Ag-(CH_2_)_14_-H

1.0

+8.7763265250 +0.0000000000 +0.0000000000

+0.0000000000 +10.1340289640 +0.0000000000

+0.0000000000 +0.0000000000 +51.5600776750

Ag S H C H

60 4 112 56 4

Cartesian

+0.0000000000 +1.6890048611 +0.0000000000

+2.9254421457 +1.6890048611 +0.0000000000

+5.8508843792 +1.6890048611 +0.0000000000

+1.4627211168 +4.2225121021 +0.0000000000

+4.3881632625 +4.2225121021 +0.0000000000

+7.3136054082 +4.2225121021 +0.0000000000

+0.0000000000 +6.7560193431 +0.0000000000

+2.9254421457 +6.7560193431 +0.0000000000

+5.8508843792 +6.7560193431 +0.0000000000

+1.4627211168 +9.2895265841 +0.0000000000

+4.3881632625 +9.2895265841 +0.0000000000

+7.3136054082 +9.2895265841 +0.0000000000

+0.0000000000 +0.0000000000 +2.3886134064

+2.9254421457 +0.0000000000 +2.3886134064

+5.8508843792 +0.0000000000 +2.3886134064

+1.4627211168 +2.5335072410 +2.3886134064

+4.3881632625 +2.5335072410 +2.3886134064

+7.3136054082 +2.5335072410 +2.3886134064

+0.0000000000 +5.0670144820 +2.3886134064

+2.9254421457 +5.0670144820 +2.3886134064

+5.8508843792 +5.0670144820 +2.3886134064

+1.4627211168 +7.6005217230 +2.3886134064

+4.3881632625 +7.6005217230 +2.3886134064

+7.3136054082 +7.6005217230 +2.3886134064

+1.4627211168 +0.8445023799 +4.7772268129

+4.3881632625 +0.8445023799 +4.7772268129

+7.3136054082 +0.8445023799 +4.7772268129

+0.0000000000 +3.3780096209 +4.7772268129

+2.9254421457 +3.3780096209 +4.7772268129

+5.8508843792 +3.3780096209 +4.7772268129

+1.4627211168 +5.9115168619 +4.7772268129

+4.3881632625 +5.9115168619 +4.7772268129

+7.3136054082 +5.9115168619 +4.7772268129

+0.0000000000 +8.4450241029 +4.7772268129

+2.9254421457 +8.4450241029 +4.7772268129

+5.8508843792 +8.4450241029 +4.7772268129

+0.0007199221 +1.6939562463 +7.1837222854

+2.9059486958 +1.6972771676 +7.2314303097

+5.8321855380 +1.7110323892 +7.1939110724

+1.4736214021 +4.2337107108 +7.1967793595

+4.3834728425 +4.2323487987 +7.1864199087

+7.3079708311 +4.2106239741 +7.1655860281

+0.0006999998 +6.7609174233 +7.1835866824

+2.9060469907 +6.7643591423 +7.2312436622

+5.8329179224 +6.7777491334 +7.1940312074

+1.4737354065 +9.3006491876 +7.1967953431

+4.3836230055 +9.2991648564 +7.1863554586

+7.3081179223 +9.2779320389 +7.1653746318

+8.7696331718 +10.0958599546 +9.5194814563

+2.8942695993 +0.0439853340 +9.6116585007

+5.9181189391 +0.0390163155 +9.5100351344

+1.4909757101 +2.5841740416 +9.6184778366

+4.3960161439 +2.5200517840 +9.6048824753

+7.3107826783 +2.5475305076 +9.6119431123

+8.7698175624 +5.0289887678 +9.5196526357

+2.8940836290 +5.1109781291 +9.6118987707

+5.9181658925 +5.1054676495 +9.5105197992

+1.4908910186 +7.6511968335 +9.6182628311

+4.3957797097 +7.5865305813 +9.6042245687

+7.3094171697 +7.6149204554 +9.6111954912

+4.3109584446 +4.2164537769 +11.5275031181

+0.0263007198 +1.7292207417 +11.5764124922

+4.3112926471 +9.2827055719 +11.5274293872

+0.0230791936 +6.7988185903 +11.5746238731

+2.4354917817 +4.3313693077 +13.0576654645

+3.5002091234 +3.0681228720 +15.7780277539

+2.5546308531 +4.5664378382 +15.6870386537

+3.7130418872 +3.2467373620 +18.2806384297

+3.9306279094 +3.3379691604 +20.7952445575

+5.7947756563 +4.6359122818 +21.9185566430

+4.1172051514 +3.3846753798 +23.3342375709

+3.3432509302 +2.7957244297 +13.2243787885

+5.2701698606 +4.0882214227 +14.2823823015

+4.3201555960 +5.5806482141 +14.1692636159

+5.4300398634 +4.3685278531 +16.8024702968

+5.6166393973 +4.5431238966 +19.3500991076

+2.7296585372 +4.7217408187 +18.2625924025

+4.5997415381 +5.9969277754 +19.3719157233

+2.9104821073 +4.7878937330 +20.8211962914

+4.7593787050 +6.0760881212 +21.9610096642

+3.0765854960 +4.8197067807 +23.3758625372

+5.9533175376 +4.6825269909 +24.4952137775

+4.9097571107 +6.1166472413 +24.5391352297

+4.2615582579 +3.4117211776 +25.8826243149

+3.2207893172 +4.8469767433 +25.9276037798

+6.0885899545 +4.6966768316 +27.0684849617

+5.0513591898 +6.1346438580 +27.1102558431

+4.3856267286 +3.4316303896 +28.4437783652

+3.3505050029 +4.8700431106 +28.4857487841

+6.2145438434 +4.7042274939 +29.6692680878

+5.1711174310 +6.1517176700 +29.7141475262

+4.4398602139 +5.8397207123 +16.7667582402

+8.3804676842 +3.6293717445 +13.0205819098

+7.1131835989 +2.3853236186 +15.7011480689

+8.3669530190 +3.6412873357 +15.6603320802

+7.1966311055 +2.3048698666 +18.2883162409

+7.3153339057 +2.2410787892 +20.8664809921

+0.2829616684 +0.8241736191 +22.0436052994

+7.4527476586 +2.1829592210 +23.4365023446

+7.0562944849 +2.4283152096 +13.0960354431

+8.5916705963 +0.8429152896 +14.3624556178

+1.0961738023 +2.0712808030 +14.3367374510

+8.7204057909 +0.8429769045 +16.9204000533

+0.1111295325 +0.8362704068 +19.4846245063

+8.3897725211 +3.6174104488 +18.2357610537

+1.2817228977 +2.1681880630 +19.4460616930

+8.4632488922 +3.5927684426 +20.8108440590

+1.4123748420 +2.1913292195 +22.0015724929

+8.5662139836 +3.5628678888 +23.3826251570

+0.4499090764 +0.8053284802 +24.6075822640

+1.5562784250 +2.1910504324 +24.5546749215

+7.6070059027 +2.1429945630 +26.0086221923

+8.6966604741 +3.5416776342 +25.9391986101

+0.6337319561 +0.8015264966 +27.1733303173

+1.7200710069 +2.2014382174 +27.1015669388

+7.7861830339 +2.1302120056 +28.5777391810

+0.0878263670 +3.5356173836 +28.4971121096

+0.8189810767 +0.8028948945 +29.7729280772

+1.9069893848 +2.2157865837 +29.6865773215

+1.1731915967 +2.1216183490 +16.8980497908

+2.4355102997 +9.4016859631 +13.0567255243

+3.4925279947 +8.1379882590 +15.7777807812

+2.5544932403 +9.6409749113 +15.6846679213

+3.6966895723 +8.3208403945 +18.2803883633

+3.9126269616 +8.4105436774 +20.7926438672

+5.7860626948 +9.6992649338 +21.9169758110

+4.1087412621 +8.4428641360 +23.3270897974

+3.3386588052 +7.8632944592 +13.2229268567

+5.2689178299 +9.1483453730 +14.2825978227

+4.3255965674 +0.5107854619 +14.1673522839

+5.4271553482 +9.4271534873 +16.8050158179

+5.6099543816 +9.6036709426 +19.3515072134

+2.7263876880 +9.8044436896 +18.2585222500

+4.6031194585 +0.9306473011 +19.3710871529

+2.9031142057 +9.8678710568 +20.8183269731

+4.7528562269 +1.0067202137 +21.9675964641

+3.0681649618 +9.8780556546 +23.3756784678

+5.9459319955 +9.7374882616 +24.4919830231

+4.8992943252 +1.0352585513 +24.5447506378

+4.2682114155 +8.4577190010 +25.8821515089

+3.2142958009 +9.8837401354 +25.9282663268

+6.0843755625 +9.7579907196 +27.0666246741

+5.0355407389 +1.0539354653 +27.1064414285

+4.3932843368 +8.4835630043 +28.4481042557

+3.3463071104 +9.9137991854 +28.4855286225

+6.2125905842 +9.7779559727 +29.6675439188

+5.1545974006 +1.0814858365 +29.7078107927

+4.4475391485 +0.7713231380 +16.7638162222

+8.3853065118 +8.6971303682 +13.0229907967

+7.1225545215 +7.4758870504 +15.7014084473

+8.3994879146 +8.7083237072 +15.6618793982

+7.2109581075 +7.4287065584 +18.2821120167

+7.3168363251 +7.3617621765 +20.8587433712

+0.2178284244 +5.8735887384 +22.0457269966

+7.4415885594 +7.2946990237 +23.4334948453

+7.0563360847 +7.5012617468 +13.0969996166

+8.5803243859 +5.9077809521 +14.3617394483

+1.0972106375 +7.1229971576 +14.3297252805

+8.6884216098 +5.9026908320 +16.9223536646

+0.0558780811 +5.8899784021 +19.4843997044

+8.4586865188 +8.6893603042 +18.2334258978

+1.2834660517 +7.1698189049 +19.4500349126

+8.5211843197 +8.6633305441 +20.8068584650

+1.4040358398 +7.1923102675 +22.0070476575

+8.6044691133 +8.6332892287 +23.3763054383

+0.3902368138 +5.8584550862 +24.6098184246

+1.5420518242 +7.2061709842 +24.5555421620

+7.5967006523 +7.2359191222 +26.0157833715

+8.7142182174 +8.6119536521 +25.9335393760

+0.6104589812 +5.8629904696 +27.1718866351

+1.7152750078 +7.2488504472 +27.0963655582

+7.7845985560 +7.2081028342 +28.5799681232

+0.0969803389 +8.6044414991 +28.4933332715

+0.8117957226 +5.8694310503 +29.7731631911

+1.9074375918 +7.2762505307 +29.6844185010

+1.1786321292 +7.1440613448 +16.8909984346

+3.5932918079 +4.1707799111 +15.6625837088

+3.7784484260 +4.3554966066 +18.2092962970

+4.5606141294 +4.8858713491 +19.4152019553

+3.4485988821 +3.8934733559 +13.1301826514

+4.2323021979 +4.4817376241 +14.3028851664

+4.3804550149 +4.7321036740 +16.8520061259

+3.9682019084 +4.4496779141 +20.7591035211

+4.7339447352 +4.9638478377 +21.9828288578

+4.1392111759 +4.4971126689 +23.3152882112

+4.8901200801 +5.0042365034 +24.5511559462

+4.2836507291 +4.5242875742 +25.8732692544

+5.0259322411 +5.0219869645 +27.1173169957

+4.4111413528 +4.5430697808 +28.4358881265

+5.1601808117 +5.0444616059 +29.6717821572

+8.2138758051 +2.5399950463 +15.6656535958

+8.2888089586 +2.5096226522 +18.2381941738

+0.1928794344 +1.9457311289 +19.4911623241

+8.1563705931 +2.5484666892 +13.1061427651

+8.7708974894 +1.9386685215 +14.3666794193

+0.0754535897 +1.9474168233 +16.9284217702

+8.3996529095 +2.4820206988 +20.8086068673

+0.3310591850 +1.9356528371 +22.0535677376

+8.5304141190 +2.4508556209 +23.3755351307

+0.4796736368 +1.9175909573 +24.6145903098

+8.6793958601 +2.4291531925 +25.9379632306

+0.6476347105 +1.9142649690 +27.1722114636

+0.0784420166 +2.4240031803 +28.4993322865

+0.8426234472 +1.9094723840 +29.7197438571

+3.5912456074 +9.2400881292 +15.6614658863

+3.7720561008 +9.4288557001 +18.2078706609

+4.5562618613 +9.9539062265 +19.4147621479

+3.4473448328 +8.9607679425 +13.1291566058

+4.2327892840 +9.5464604982 +14.3021050625

+4.3800359453 +9.7984282313 +16.8512554111

+3.9583931348 +9.5219312838 +20.7577304762

+4.7257811718 +10.0284686620 +21.9835001700

+4.1307173593 +9.5553192609 +23.3137883285

+4.8821318677 +10.0567888140 +24.5514250898

+4.2799931073 +9.5703349530 +25.8730748729

+5.0191719245 +10.0751124558 +27.1160274782

+4.4095878553 +9.5950181033 +28.4363996025

+5.1548566532 +10.1078806358 +29.6698682471

+8.2258912105 +7.6100636207 +15.6651354171

+8.3110972297 +7.5867691363 +18.2353733219

+0.1858287969 +6.9947473728 +19.4909339130

+8.1568623307 +7.6169272971 +13.1062768213

+8.7707491695 +7.0017284013 +14.3645489569

+0.0763472830 +7.0027056258 +16.9262294357

+8.4107651431 +7.5563124270 +20.8056452564

+0.3128281219 +6.9820825728 +22.0545277862

+8.5283931066 +7.5233485589 +23.3733170162

+0.4569067926 +6.9689558637 +24.6151084885

+8.6742927773 +7.4999513155 +25.9380431487

+0.6395411822 +6.9754455945 +27.1702738359

+0.0791787015 +7.4929279268 +28.4981041255

+0.8414890192 +6.9759070982 +29.7187729808

+4.6943176902 +4.6813565106 +30.6071943672

+0.4136219502 +2.2955270013 +30.6636196538

+4.6934659477 +9.7439074595 +30.6071856020

+0.4153223634 +7.3656784720 +30.6623842743

**Supplementary Table** **12**. Summary of the parameters used for fitting of current across Ag-S(CH_2_)_14_X//GaO*_x_*/EGaIn junctions using Landauer theory.

| Parameters | X=H | X=F | X=Cl | X=Br | X=I |
| --- | --- | --- | --- | --- | --- |
| $\Gamma$ (meV) | 0.497 | 0.529 | 1.74 | 5.99 | 14.4 |
| $\eta$ | 0.470 | 0.460 | 0.450 | 0.480 | 0.480 |
| $\delta E_{ME}$(eV) | 4.300 | 3.600 | 2.600 | 2.200 | 1.700 |
| $\sigma$ (eV) | 0.190 | 0.190 | 0.190 | 0.190 | 0.190 |

**References**

1. Wang, D*.* et al. Tuning the tunneling rate and dielectric response of SAM-based junctions via a single polarizable atom. *Adv. Mater.* **27**, 6689-6695 (2015).

2. Kim, K.-Y.; Kim, B. C.; Lee, H. B. & Shin, H. Nucleophilic fluorination of triflates by tetrabutylammonium bifluoride. *J. Org. Chem.* **73**, 8106-8108 (2008).

3. Kaiser, E. & Gunther, E. P. Alcoholysis of esters with aluminum alcoholates^1^. *J. Am. Chem. Soc.* **78**, 3841-3843 (1956).

4. Alvarado, R. J., Mukherjee, J., Pacsial, E. J., Alexander, D. & Raymo, F. M. Self-assembling bipyridinium multilayers. *J. Phys. Chem. B* **109**, 6164-6173 (2005).

5. Bérubé, M., Kamal, F., Roy, J. & Poirier, D. A Dehydrohalogenation Methodology for Synthesizing Terminal Olefins under Mild Conditions. *Synthesis* **2006**, 3085-3091 (2006).

6. Ismaili, H., Alizadeh, A., Snell, K. E. & Workentin, M. S. Remarkable high-yielding chemical modification of gold nanoparticles using uncatalyzed click-type 1,3-dipolar cycloaddition chemistry and hyperbaric conditions. *Can. J. Chem.* **87**, 1708-1715 (2009).

7. Paquette, L. A., Maynard, G. D., Ra, C. S. & Hoppe, M. Cleavage of carbon-carbon bonds with high stereochemical control. 7. Chiral .alpha.-silyl benzoylcycloalkanes undergo base-catalyzed cleavage with retention of configuration when not sterically congested. *J. Org. Chem.* **54**, 1408-1418 (1989).

8. Effenberger, F. & Heid, S. Synthesis of Model Compounds for the Formation of Self-Assembled Monolayers on a Silicon Surface. *Synthesis* **1995**, 1126-1130 (1995).

9. Chen, X. et al. Molecular diodes with rectification ratios exceeding 10^5^ driven by electrostatic interactions. *Nat. Nanotechnol.* **12**, 797-803 (2017).

10. Yu, X*.* et al. New soft X-ray facility SINS for surface and nanoscale science at SSLS. *J. Electron Spectros. Relat. Phenomena* **144–147**, 1031-1034 (2005).

11. Yuan, L., Breuer, R., Jiang, L., Schmittel, M. & Nijhuis, C. A. A molecular diode with a statistically robust rectification ratio of three orders of magnitude. *Nano Lett.* **15**, 5506-5512 (2015).

12. Qi, Y. et al. Filled and empty states of alkanethiol monolayer on Au (111): Fermi level asymmetry and implications for electron transport. *Chem. Phys. Lett.* **511**, 344-347 (2011).

13. Nerngchamnong, N. et al. The role of van der Waals forces in the performance of molecular diodes. *Nat. Nanotechnol.* **8**, 113-118 (2013).

14. Chen, X., Hu, H., Trasobares. J. & Nijhuis, C. A. Rectification ratio and tunneling decay coefficient depend on the contact geometry revealed by in situ imaging of the formation of EGaIn junctions. *ACS Appl. Mater. Inter.* **11**, 21018-21029 (2019).

15. Jiang, L., Sangeeth, C. S. S., Wan, A., Vilan, A. & Nijhuis, C. A. Defect scaling with contact area in EGaIn-based junctions: impact on quality, joule heating, and apparent injection current. *J. Phys. Chem. C* **119**, 960-969 (2015).

16. Sangeeth, C. S. S., Wan, A. & Nijhuis, C. A. Equivalent circuits of a self-assembled monolayer-based tunnel junction determined by impedance spectroscopy. *J. Am. Chem. Soc.* **136**, 11134-11144 (2014).

17. Sangeeth, C. S. S., Wan, A. & Nijhuis, C. A. Probing the nature and resistance of the molecule-electrode contact in SAM-based junctions. *Nanoscale* **7**, 12061-12067 (2015).

18. Macdonald, J. R. & Johnson, W. B. Impedance spectroscopy: theory, experiment, and applications, 2 edn. *John Wiley & Sons, Inc.* 1-27 (2005).

19. Kresse, G. & Furthmüller, J. Efficient iterative schemes for ab initio total-energy calculations using a plane-wave basis set. *Phys. Rev. B* **54**, 11169-11186 (1996).

20. Blöchl, P. E. Projector augmented-wave method. *Phys. Rev. B* **50**, 17953-17979 (1994).

21. Perdew, J. P., Burke, K. & Ernzerhof, M. Generalized gradient approximation made simple. *Phys. Rev. Lett.* **77**, 3865-3868 (1996).

22. Schreiber, F. Self-assembled monolayers: from  simple  model systems to biofunctionalized interfaces. *J*. *Phys: Condens*. *Matter* **16**, R881-R900 (2004).

23. Bučko, T., Hafner, J. & Ángyán, J. G. Geometry optimization of periodic systems using internal coordinates. *J. Chem. Phys.* **122**, 124508 (2005).

24. Heitzer, H. M., Marks, T. J. & Ratner, M. A. First-principles calculation of dielectric response in molecule-based materials. *J. Am. Chem. Soc.* **135**, 9753-9759 (2013).

25. Romaner, L., Heimel, G., Ambrosch-Draxl, C. & Zojer, E. The dielectric constant of self-assembled monolayers. *Adv. Funct. Mater.* **18**, 3999-4006 (2008).

26. Bondi, A. van der Waals volumes and radii. *J. Chem. Phys.* **68**, 441-451 (1964).
